# The MBO2/FAP58 heterodimer stabilizes assembly of inner arm dynein *b* and reveals axoneme asymmetries involved in ciliary waveform

**DOI:** 10.1091/mbc.E23-11-0439

**Published:** 2024-04-19

**Authors:** Gang Fu, Katherine Augspurger, Jason Sakizadeh, Jaimee Reck, Raqual Bower, Douglas Tritschler, Long Gui, Daniela Nicastro, Mary E. Porter

**Affiliations:** a Department of Cell Biology, University of Texas Southwestern Medical Center, Dallas, TX 75390; b Department of Genetics, Cell Biology, and Genetics, University of Minnesota, Minneapolis, MN 55455; University of California, San Francisco

## Abstract

Cilia generate three-dimensional waveforms required for cell motility and transport of fluid, mucus, and particles over the cell surface. This movement is driven by multiple dynein motors attached to nine outer doublet microtubules that form the axoneme. The outer and inner arm dyneins are organized into 96-nm repeats tandemly arrayed along the length of the doublets. Motility is regulated in part by projections from the two central pair microtubules that contact radial spokes located near the base of the inner dynein arms in each repeat. Although much is known about the structures and protein complexes within the axoneme, many questions remain about the regulatory mechanisms that allow the cilia to modify their waveforms in response to internal or external stimuli. Here, we used *Chlamydomonas mbo* (move backwards only) mutants with altered waveforms to identify at least two conserved proteins, MBO2/CCDC146 and FAP58/CCDC147, that form part of a L-shaped structure that varies between doublet microtubules. Comparative proteomics identified additional missing proteins that are altered in other motility mutants, revealing overlapping protein defects. Cryo-electron tomography and epitope tagging revealed that the L-shaped, MBO2/FAP58 structure interconnects inner dynein arms with multiple regulatory complexes, consistent with its function in modifying the ciliary waveform.

## Abstract (executive summary)

Cells alter the shape of their ciliary waveforms in response to internal or external stimuli, but the identity and location of the polypeptides responsible for regulating the waveform are poorly characterized.

Using motility mutants available in *Chlamydomonas*, the authors applied proteomic analyses to identify axonemal proteins involved in waveform conversion. Cryo-electron tomography and classification averaging revealed defects in assembly of an inner arm dynein and other doublet microtubule-specific structures.

These findings provide new insights into the identity of conserved proteins recently linked to human disease and the complexity of their structural organization within the axoneme.

## INTRODUCTION

Eukaryotic cilia and flagella are microtubule-based extensions of the cell surface important for cell motility and signaling. Defects in cilia or flagella have been linked to a complex group of diseases collectively termed ciliopathies (reviewed in Reiter and Leroux, 2017). In vertebrate embryos, motility is required for the rotary movement of nodal cilia that determines the left-right body axis (reviewed in Babu and Roy, 2013). In adults, motility is essential for the circulation of cerebrospinal fluid, the clearance of particles and mucus in the respiratory tract, and sperm motility. Defects in the assembly, beating, or signaling of motile cilia have been linked to situs inversus, hydrocephalus, curvature of the spine, chronic respiratory disease, and infertility (Mitchison and Valente, 2017). Given the complex structure of cilia and the large number of genes involved in their assembly and motility, determining the underlying basis for disease can be challenging. The study of model organisms with motile cilia like *Chlamydomonas* and *Tetrahymena* has proven useful for understanding the network of genes and proteins required for motility.

**FIGURE 1:**
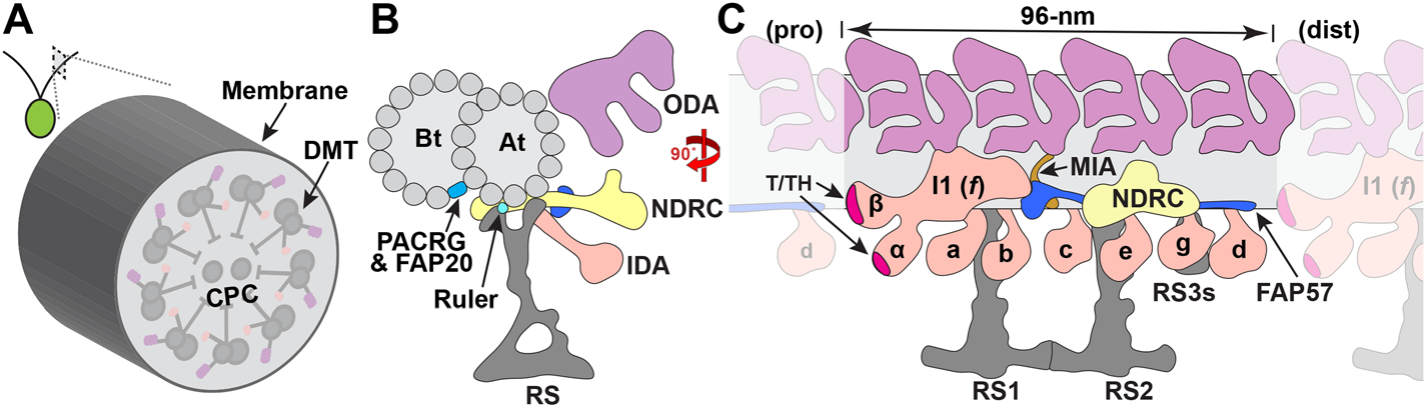
Schematic diagram of structures in the 96-nm axoneme repeat. (A) Drawing of the biflagellate green *alga Chlamydomonas* and diagram of a cross-section through the axoneme showing the nine outer DMTs surrounding the inner CPC. (B) Diagram of a single DMT shown in cross-section at higher magnification with the complete A-tubule (At) and associated B-tubule (Bt). The inner junction between the At and Bt is composed of alternating subunits of PACRG and FAP20 (aqua). The A-tubule contains a multisubunit ODA with three motor domains (lilac), multiple IDA isoforms (pink), the NDRC (yellow), and one of three radial spokes (RS, grey). The small light blue dot indicates the location of the ruler subunits FAP59 (CCDC39) and FAP172 (CCDC40) that determine the spacing of the RSs and IDAs. Also shown in cross-section is a portion of the FAP57 complex (dark blue). (C) Diagram of a single DMT shown in longitudinal view from the proximal (pro) to distal (dist) region of the 96-nm repeat, containing four ODAs (lilac), the seven IDA isoforms I1 (*f*), *a, b, c, e, g, d* (pink), and the three RS structures (RS1, RS2, RS3S), with I1 *(f*) above RS1 and the N-DRC (yellow) above RS2. The two I1 motor domains (α, β) are also attached to the DMT through the Tether-Tether Head (T/TH) (fuschia). The MIA complex (brown) links the base of I1 to the ODAs, the DMT, and the proximal portion of the FAP57 complex (dark blue). The coiled-coil domains of the FAP57 extend beyond the N-DRC to contact IDA *g* and *d*.

Comparative genomics and proteomics have identified >1000 proteins as structural components of the ciliary axoneme and ciliary membrane (reviewed in van Dam *et al.*, 2019; Sakoto-Antoku and King, 2022). Most motile cilia contain nine outer doublet microtubules (DMTs) surrounding two singlet central pair (CP) MTs. Each DMT contains thousands of large, multisubunit motors known as the outer and inner dynein arms (ODA, IDA). The dynein motors generate the force for microtubule sliding between the A-tubule of one DMT and the B-tubule of the adjacent DMT. The ODAs and IDAs are composed of different heavy, intermediate, and light chain (DHC, IC, LC) subunits attached in two rows on the A-tubule. They are further organized into functional units that repeat every 96 nm, with four identical ODAs and seven unique IDAs (I1/*f, a, b, c, e, g, d*) bound at discrete sites within the repeat (Figure 1). Motor activity is coordinated in part by signals from the CP and its numerous projections that contact three radial spokes (RS1, RS2, RS3, or RS3S) bound near the base of the IDAs in each 96-nm repeat (reviewed in Brown *et al.*, 2023; Witman and Mitchell, 2023). The IC/LC complex of the I1/*f* dynein forms a regulatory node at the base of RS1, the nexin-dynein regulatory complex (N-DRC) forms another at the base of the RS2, and a calmodulin spoke complex (CSC) connects between the bases of RS2, RS3/RS3S and the N-DRC (Heuser *et al.*, 2009; 2012a,b). The I1 dynein and N-DRC also connect to the ODAs and other structures in the 96-nm repeat to stabilize the binding of the IDAs and coordinate dynein activity (Heuser *et al.*, 2009, 2012a,b); Oda *et al.*, 2013). This network of regulatory proteins includes the tether/tether head (T/TH) structures associated with the I1 dynein motor domains (Fu *et al.*, 2018; Kubo *et al.*, 2018), the MIA complex located between the I1 dynein and the ODAs (King and Dutcher, 1997; Yamamoto *et al.*, 2013), and the FAP57 complex linking the MIA complex, IDAs *g* and *d,* and other structures (King *et al.*, 1994; Lin *et al.*, 2019; Bustamante-Marin *et al.*, 2020; Ghanaeian *et al.*, 2023).

Many mutations that disrupt the assembly of ODAs, IDAs, RSs, CP, and its projections, MIA complex, N-DRC, T/TH, and FAP57 complex have been characterized over decades of work in *Chlamydomonas*, *Tetrahymena*, and other organisms, and their motility phenotypes are very diverse, ranging from flagellar paralysis, ciliary twitching, reduced beat frequency, and/or altered ciliary waveforms with reduced amplitudes (reviewed in Sale and Dutcher, 2023). However, ciliary motility is extremely complex, and many organisms can adjust the pattern of their waveforms in response to internal and external stimuli. For instance, *Chlamydomonas* can adjust the beat frequency of its *cis* and *trans* cilia to undergo both positive and negative phototaxis, or it can reverse its swimming direction in response to bright light (reviewed in Witman, 1993; Dutcher, 2020; Sale and Dutcher, 2023). The latter involves the conversion of an asymmetric ciliary-type waveform to a more symmetric, flagellar-type waveform. The structures and polypeptides responsible for waveform conversion are poorly understood. A handful of mutations result in cells that swim forward with a symmetric waveform (*pf10, pf12/pacrg*, *fap20*; McVittie, 1972; Dutcher *et al.*, 1988; Frey *et al.*, 1997; Dymek *et al.*, 2019), and a second group result in cells that swim backwards with a symmetric waveform (*mbo1, mbo2*, *mbo3*; Segal *et al.*, 1984; Table 1). Characterization of *fap20* and *pf12/pacrg* mutants by conventional transmission electron microscopy (TEM) and cryo-electron tomography (cryo-ET) has shown that FAP20 and PACRG form the inner junction between the A- and B- tubules of the DMT (Yanagisawa *et al.*, 2014; Dymek *et al.*, 2019; Figure 1). These studies also revealed secondary defects in the assembly of structures located inside the lumen of the proximal B-tubule (beak-MIP), IDA *b*, and the I1 dynein T/TH complex (Yanagisawa *et al.*, 2014; Dymek *et al.*, 2019). How defects in the inner DMT junction might lead to changes in waveform asymmetry remains unclear, but the structural defects suggested that *fap20* and *pacrg* mutants may have additional polypeptide deficiencies. Previous studies of *mbo1*, *mbo2*, and *mbo3* mutants described defects in the assembly of eight axonemal polypeptides ranging in size from ∼33-245 kD, but the identities and locations of the missing proteins were unknown (Segal *et al.*, 1984). The only structural defect identified by conventional TEM was the absence of beak structures located within the lumens of the B-tubules of DMT5 and DMT6. The isolation and characterization of new alleles of *MBO2* by insertional mutagenesis later revealed MBO2 to be a conserved, coiled-coil protein found along the length of the axoneme (Tam and Lefebvre, 1993, 2002), but the *MBO1* and *MBO3* gene products remain unknown (Table 1)

**TABLE 1:** Motility mutants compared in this study.

Strain name	Gene product	Motility phenotypes	Biochemical phenotypes	Structural phenotypes	References
Motility mutants					
*mbo2-4*	MBO2	Reduced swimming velocityMoves backwards only (*mbo*)Symmetric waveform	eight spots reduced by two-dimensional PAGE>24 proteins reduced (TMT)Including MBO2 and DHC5	Defects in DMT5 and DMT6 beaksIDA *b* reduced, DMT specific defects in L-shaped structure	Segal *et al.*, 1984; Tam and Lefebvre, 2002This study
*MBO2-HA*	MBO2	Recovery of WT motility	Recovery of missing proteins including MBO2 and DHC5	Rescue of DMT defectsMBO2-HA along length of axonemeRecovery of IDA *b*	Tam and Lefebvre, 2002This study
*MBO2-N-SNAP*	MBO2	Recovery of WT motility	Recovery of MBO2, DHC5	Rescue of structural defectsN-SNAP tag near IDA *b*, L-structure	This study
*MBO2-C-SNAP*	MBO2	Recovery of WT motility	Recovery of MBO2, DHC5	Rescue of structural defectsC-SNAP tag near base of L-structure	This study
*MBO2-M-SNAP*	MBO2	Recovery of near WT motility	Recovery of MBO2, DHC5	Rescue of structural defectsM-SNAP tag in middle of L-structure	This study
*mbo1*	Unknown	Moves backwards only (*mbo*)Symmetric waveform	six spots reduced (two-dimensional PAGE)19 proteins reduced (iTRAQ)	Defects in DMT5 and DMT6 beaks	Segal *et al.*, 1984This study
*mbo3*	Unknown	Mixed phenotypeSome *mbo*, some paralyzed	six spots reduced (two-dimensional PAGE)MBO2, DHC5 reduced (Western)	Defects in DMT5 and DMT6 beaks	Segal *et al.*, 1984This study
*ida8-1*	FAP57	Reduced swimming velocityforward movementSuppressor of *pf10*	FAP57, FAP337, DHC2, DHC3, DHC7 reduced, FBB7, FAP331, Cre07.g313850 increased (iTRAQ)FAP331, FAP337 reduced in *mbo2*	IDA *d, g* reduced on DMTs5-9I1 distal structure reducedN-SNAP tag distal to I1(*f)* IDAC-SNAP tag distal to N-DRC	Li *et al.*, 2019
*pf10*	unknown	Reduced swimming velocitySymmetric waveform, partial suppression by light	uncharacterized	Not analyzed	Dutcher *et al.*, 1988
*pf12*	PACRG	Reduced swimming velocity Symmetric waveformTwitch and jerk	Lacks subunit of DMT junction10 proteins reduced in both *pf12* and *mbo2* (iTRAQ)	Gap every 8 nm in inner DMT junctionIDA *b* slightly reducedDMT beaks reducedI1 tether base reduced	[Bibr B55][Bibr B17]This study
*pf12; fap20*	PACRG; FAP20	Reduced swimming velocitySymmetric waveformTwitch and jerk	Lacks both subunits of DMT junction62 proteins reduced in *pf12; fap20*21 also reduced in *mbo2* (TMT)	Inner junction of DMT missingIDA *b* significantly reducedDMT beaks reducedI1 tether base missing	Yanagisawa *et al.*, 2014; Dymek *et al.*, 2019This study

Dynein mutants					
*dhc5-264*	DHC5	WT	Lacks DHC5 motor domain	Not analyzed	Li *et al.*, 2016 This study
*dhc5-298*	DHC5	WT	Lacks DHC5 motor domain	Not analyzed	Li *et al.*, 2016 This study
*dhc5-438*	DHC5	WT	Lacks DHC5 motor domain	Not analyzed	Li *et al.*, 2016 This study
*dhc5-953*	DHC5	WT	Lacks DHC5 motor domain	Not analyzed	Li *et al.*, 2016 This study
*pf28*	DHC15(γDHC)	Reduced beat frequency	Lacks ODA subunits	ODA missing	Mitchell and Rosenbaum, 1985
*pf9-2; pf28*	DHC1/1αDHC DHC15/γDHC	Short flagellaParalyzed	ODA and I1(*f*) IDA missingDHC3, DHC4, DHC11 increasedeight MBO2-related proteins altered (including DHC5)	Not analyzed	[Bibr B96][Bibr B71][Bibr B32]This study

Additional details on all strains used in this study are provided in Supplemental Table 1. Biochemical defects in axonemes were determined by iTRAQ or TMT based proteomics or Western blots.

Here, we reanalyzed the *mbo1* and *mbo2* mutants to identify the polypeptides and structures responsible for waveform conversion and backwards swimming. We used proteomic approaches and tagged *MBO2* transgenes to identify a large group of polypeptides missing or reduced in the *mbo2* mutant and restored in *MBO2* rescued strains. Comparative proteomics revealed that several proteins reduced in *mbo2* were also altered in *pf12(pacrg), pf12; fap20* and *ida8 (fap57)*, three other mutants with defects in waveform asymmetry (Table 1). Cryo-ET of *mbo2* revealed several DMT-specific structural defects, including changes in an elongated, L-shaped structure reaching from the base of IDA *b* and extending to other axonemal structures beyond the N-DRC (Lin *et al.*, 2019; Walton *et al.*, 2023). Rescue with SNAP-tagged *MBO2* constructs followed by streptavidin gold labeling and classification averaging shows that MBO2 is one of the components of the L-shaped structure that interconnects multiple regulatory components and stabilizes the assembly of IDA *b* within the 96-nm axoneme repeat.

## RESULTS

### Identification of polypeptide defects in mbo2 and mbo1 axonemes

**FIGURE 2:**
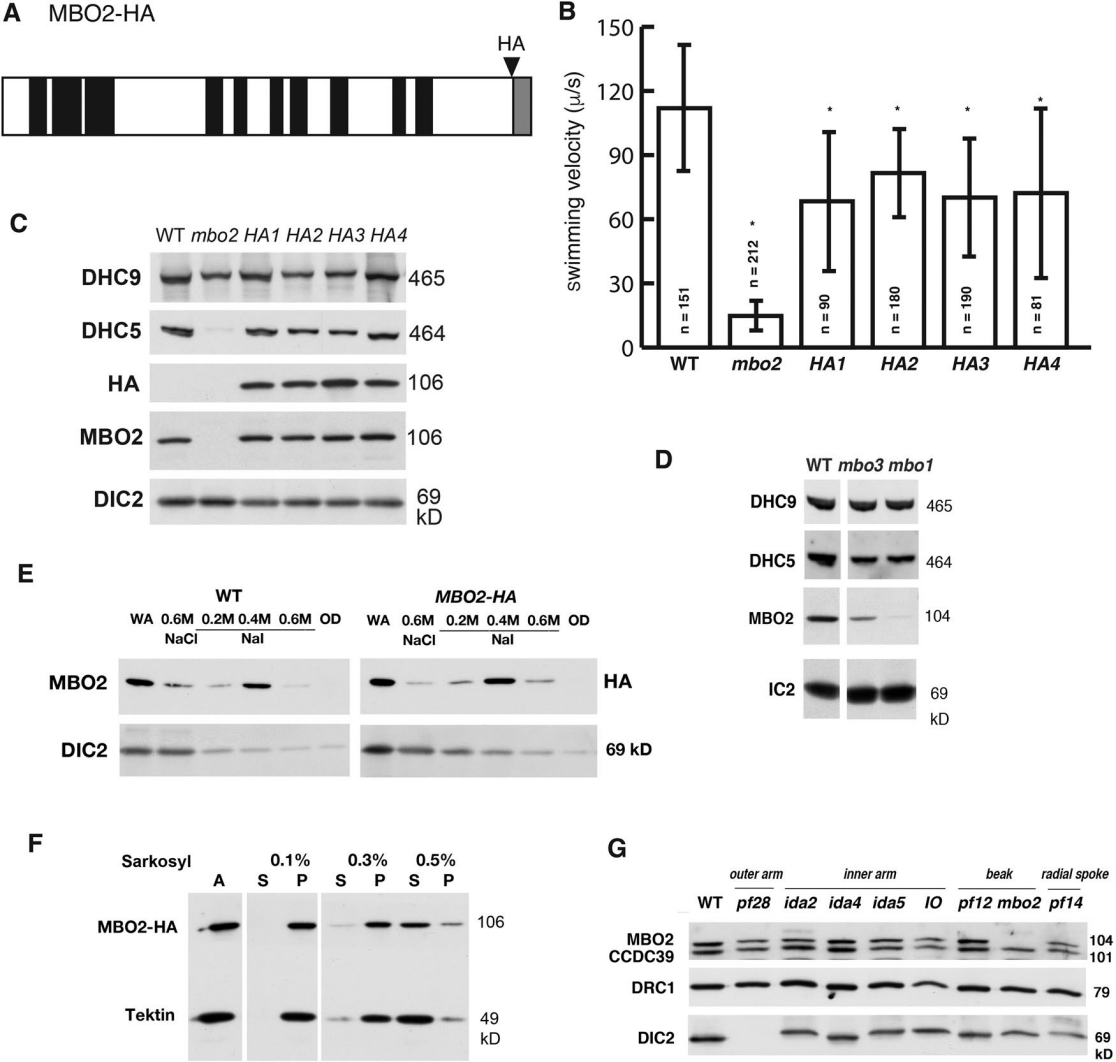
MBO2 is involved in stabilizing the assembly of DHC5. (A) A schematic diagram of the MBO2 polypeptide showing the location of coiled-coil domains (black) and a disordered region in the C-terminus gray). Also shown is the position of the HA epitope tag. (B) Transformation of a *mbo*2 mutant with *MBO2-HA* restores forward swimming. The forward swimming velocities of WT, *mbo2*, and *HA*-rescued strains (*HA1-HA4*) were measured by phase contrast light microscopy. All the *HA*-rescued strains swam forwards significantly faster (*P* < 0.5) than *mbo2*, but significantly slower than WT, consistent with previous reports (Tam and Lefebvre, 2002). (C) Western blots of axonemes probed with different antibodies show the recovery of MBO2 and DHC5 in the *HA*-rescued strains. DHC9 and DIC2 are loading controls for other IDA and ODA isoforms. (D) Western blots of *mbo1* and *mbo3* axonemes show that MBO2 and DHC5 are also reduced in these two strains. (E) WT and *mbo2; MBO2-HA* axonemes were subjected to sequential extraction with 0.6 M NaCl and 0.2–0.6 M NaI buffers, and analyzed on Western blots (WA, whole axoneme; OD, extracted outer doublets). The majority of MBO2 was extracted with 0.4 M NaI, whereas dynein subunits (DIC2) were largely extracted with 0.6 M NaCl. (F) Western blot of WT axonemes (A) that were extracted with increasing concentrations of Sarkosyl (S, supernatant; P, pellet). (G) Western blot of axonemes from different motility mutants lacking outer arms (*pf28*), inner arms (*ida2, ida4, ida5*), B-tubule beaks (*pf12, mbo2*), and radial spokes (*pf14*).

Transformation of *mbo2* with an HA-tagged *MBO2* transgene rescued the *mbo* motility phenotype, increased forward swimming velocities, and restored assembly of the missing MBO2 subunit along the length of the axoneme (Figure 2, A–C; Supplemental Figure S1; Supplemental Tables S2 and S3, see also Tam and Lefebvre, 2002). The forward swimming velocities observed in four independent HA-tagged transformants were not completely wild-type (Figure 2B, consistent with previous studies [Tam and Lefebvre, 2002]). To identify polypeptides associated with MBO2, we purified axonemes from wild-type (WT), *mbo2*, *MBO2-HA* rescued strains, and *mbo1,* labeled them using iTRAQ isobaric tags, and subjected them to quantitative mass spectrometry (see *Materials and Methods*). Although the *MBO1* gene product has not been identified, earlier work showed that *mbo1* and *mbo2* mutants have similar deficiencies in axonemal polypeptides (Segal *et al.*, 1984, see Table 1). The abundance of each polypeptide present in *mbo* mutant axonemes was expressed as a protein ratio relative to its abundance in WT axonemes. Nineteen proteins were significantly reduced below 0.75 (*P* value < 0.05) in four iTRAQ experiments using *mbo2* and *mbo1* (Supplemental Table S4). We later modified the protocol using three independent samples each of WT, *mbo2*, and *MBO2-HA* axonemes, TMT-based isobaric tags, and more sensitive instrumentation (Supplemental Figure S2A). Thirty-eight polypeptides were identified as significantly reduced (*P* value < 0.05) in *mbo2* axonemes relative to the *MBO2-HA* rescued samples (Supplemental Table S4). Fourteen of these were detected by a small number of peptides, showed limited sequence coverage, and were poorly represented in previously published proteomes of WT axonemes; many were also poorly conserved outside of green algae. Their characteristics are listed in Supplemental Table S4 and are not discussed further here. The remaining 24 proteins were detected by several peptides, showed broad sequence coverage, and were well represented in previous proteomes (Table 2, Supplemental Table S4). Their characteristics are summarized in Table 3.

**TABLE 2:** Protein ratios in axonemes from *mbo2* and rescued *MBO2-HA* strains.

Genome ID (Cre)	Name	Molecular Weight	Coverage (%)	Unique Peptides (N)	*mbo2*/WT	*mbo2/MBO2-HA*	Ratios in other mutants
Cre14.g618300		46.7	22	10	**0.01**	**0.01**	Unchanged
Cre08.g383101	**FAP324**	69.8	56	36	**0.09**	**0.09**	Reduced in short
Cre13.g801543Cre13.g584300*	**FAP58**	101.7	52	36	**0.11**	**0.09**	Unchanged
Cre10.g441900	**FAP145**	30.7	44	11	**0.13**	**0.12**	Unchanged
Cre09.g416550	**MBO2**	104.1	48	54	**0.13**	**0.12**	Unchanged
Cre02.g077600	**FAP51**	207.9	42	76	**0.22**	**0.20**	Reduced *pf12; fap20*
Cre16.g682850		94.6	31	31	**0.29**	**0.26**	Reduced *pf12, pf12; fap20*
Cre03.g181100		93.5	33	22	**0.18**	**0.28**	Reduced *pf12; fap20*
Cre12.g509350	**FAP238**	41.8	31	19	**0.35**	**0.31**	Reduced short, *pf12, pf12; fap20,*
Cre06.g289600		181	20	29	**0.32**	**0.32**	Unchanged
Cre06.g275950	**FAP395**	219.2	25	47	**0.42**	**0.35**	Reduced *pf12; fap20*
Cre12.g555378		95.7	25	17	**0.34**	**0.36**	Reduced *pf12; fap20*
Cre02.g107050	**DHC5**	463.6	27	108	**0.37**	**0.37**	Reduced short, *pf12, pf12; fap20*
Cre12.g486700	**FAP271**	40.5	52	18	**0.41**	**0.41**	Reduced *pf12, pf12; fap20*
Cre16.g681301	**MOT12/FAP423**	51.6	17	7	**0.36**	**0.41**	Reduced *pf12; fap20*
Cre10.g430700	**FAP308**	315	24	63	**0.40**	**0.45**	Increased short
Cre03.g144887		293	19	38	**0.31**	**0.54**	Reduced *pf12; fap20*
Cre01.g040600	**FAP327**	185.9	23	40	**0.44**	**0.37**	Increased short, reduced *pf12; fap20*
Cre05.g234640		346.6	16	44	0.52	**0.46**	Increased short
Cre12.g483800	**(CPY)**	258.5	16	35	0.55	**0.47**	Increased short, reduced *pf12; fap20*
Cre07.g325751	**FAP258**	74.4	48	32	0.52	**0.49**	Reduced *pf12*, *pf12; fap20*
Cre06.g278324	**FAP343**	114.2	27	29	0.54	**0.50**	Reduced *pf12*, *pf12; fap20,* short

FAP57 associated							
Cre06.g308000	**FAP331**	205.1	28	49	**0.17**	**0.38**	Increased *fap57*, reduced *pf12, pf12; fap20,* short
Cre13.g562800	**FAP337**	108.1	25	23	**0.44**	**0.38**	Reduced *fap57, pf12, pf12; fap20,* short

Control proteins							
Cre04.g217914	**FAP57**	145.1	40	64	1.44	1.17	Missing *fap57*
Cre03.g143827	**FBB7**	180.2	31	45	0.69	0.85	FAP57 paralogue, increased *fap57*
Cre07.g313850	**FAP337 paralogue**	135.7	28	30	0.48	0.64	Increased *fap57, reduced pf12*
Cre11.g476850	**DRC4**	55.1	68	52	1.03	1.16	
Cre02.g113400	**PACRG**	34.1	56	23	1.00	1.08	Reduced *pf12, pf12; fap20*
Cre17.g701250	**FAP59**	105.3	53	66	1.31	1.14	
Cre17.g698365	**FAP172**	101.2	64	75	1.24	1.10	
Cre16.g689600	**FAP73**	38.5	43	18	1.06	1.10	
Cre02.g103950	**FAP100**	65.1	33	25	0.98	1.05	
Cre12.g536600	**FAP78**	267	28	63	1.09	1.06	
Cre13.g584400	**FAP189**	101.9	60	46	1.19	1.22	
Cre12.g511050	**RSP16**	39	57	24	1.08	1.02	
Cre03.g178500	**RIB43**	42.6	69	41	1.16	1.14	
Cre02.g091700	**RIB72**	71.9	47	38	1.06	1.12	
Cre03.g190950	**TUA**	49.6	55	18	0.97	0.98	
Cre12.g542250	**TUB**	49.6	56	20	0.99	1.01	
Cre16.g655750	**TEK1**	53.9	38	25	1.52	1.04	

Three independent samples (biological replicates) of WT, *mbo2-4*, and *MBO2-HA* axonemes were labeled with three separate TMT reagents (nine samples total) and analyzed by mass spectrometry. The *Chlamydomonas* genome ID number is shown in the first column, and the common name of the protein is shown in the second. The predicted molecular weight is shown in the third column and the percent coverage of the protein sequence is shown in the fourth. The number of unique peptides used for protein identification is shown in the fifth column. The *mbo2*/WT ratios are shown sixth column, and *mbo2/MBO2-HA* ratios are shown in the seventh. Ratios that were significantly different from control samples (*P* < 0.05) are shown in bold. The increase or decrease in protein ratios in other mutant axonemes are noted in the last column. See Supplemental Table S4 for additional details on other proteins identified during proteomic analyses of *mbo2* and other mutants (*ida8/fap57, pf12/pacrg, pf12; fap20, short pf28 pf9-2*).

**TABLE 3:** Characteristics of proteins reduced in *mbo2* and restored in *MBO2-HA*.

Protein Name (JGI Gene ID Phytozome13)	AA (MW in kD)	Predicted Domains	*Volvox carteri* JGI ID (Blastp)	*Physcomitrella patens* GenBank ID (Blastp)	*Homo sapiens* GenBank ID (Blastp)	Comments
MBO2 pattern (significantly reduced in only *mbo2*)						
**Cre06.g289600**	1852(181)	CC	0001s1496(1e-35)	ND	ND	fraction unknownpoorly conserved
**MBO2**Cre09.g416550	920(104)	CC, low complexity	0011s0271(0.0)	PNR44978.1(1e-100)	**CCDC146**NP_065930.2(1e-102)	extracted axoneme
**FAP58**Cre13.g801543Cre13.g584300	871(102)	CC, low complexity	0001s0063(0.0)	**CFAP58L**XP_024400258.1(0.00)	**CCDC147**NP_0010088723.1(0.0)	extracted axonemeFAP189 paralogue
**Cre14.g618300**	455(46.7)	CC	0001s1452(1e-170)	ND	ND	detected in FPLC peak *b*poorly conserved
**FAP145**Cre10.g441900	280(30.7)	CCTSNAXIP1	0001s1252(1e-175)	ND	**TSNAXIP1**XP_011521536.1(8e-04)	found in salt extract, weakly related to translin associated factor X interacting protein

FAP57 associated (altered in *ida8/fap57,* enriched in high salt extracts)						
**FAP331**Cre06.g308000	1989(205)	WD, CC	0009s0155(0.0)	**CFAP57L**XP_024388298.1(4e-07)	**CFAP57**(3e-13)	FAP57 paralogueIncreased in *fap57*reduced in *pf12, pf12; fap20*
**FAP337**Cre13.g562800	1004(102)	WD, EF hand	0028s0019(0.00)	**WDR49-like**XP_024383320.1(3e-75)	**WDR49**NP_001335880(9e-48)	Reduced in *fap57*reduced in *pf12;fap20*

DHC5 pattern (detected in high salt extract and reduced in short axonemes)						
**DHC5**Cre02.g207050	4185(464)	AAA, CC	0022s0148(0.0)	**DHC7**XP_024376875.1(0.0)	**DNAH7**AAL37427.1(0.0)	Subunit of IDA *b*reduced in *pf12, pf12; fap20*
**FAP343**Cre06.g278234	1079(114.2)	CC, WW	0002s0194(2.2e-140)	**CEP164L**XP_024400159.1(7e-12)	**CEP164**BAG64184.1(5e-10)	reduced in *pf12, pf12; fap20*weakly related to CEP164
**FAP324**Cre08.g383101	631(70)	CC, Kelch, FN3	0011s0100(0.0)	XP-024360494.1(2e-22)	**KLHDC3**EAX04137.1(1e-12)	Weakly related to kelch domain protein in testis
**FAP238**Cre12.g509350	366(42)	EF-hand, CC	0016s0228(3.3e-154)	XP_02439029.1(0.095)	CAA27243.1(0.003)	reduced in *pf12, pf12 fap20*

*pf12, pf12; fap20* pattern (reduced in *pf12* or *pf12; fap20* but not altered in short axonemes)						
**Cre03.g144887**	2975(293)	IQ, LRR	0014s0054(1e-106)	XP_024395261.1(9e-13)	**LRRC74a**NP_001372035.1(5e-10)	found in salt extractweakly related to LRRC74 found in testis
**FAP395**Cre06.g275950	2136(219)	CC	0009s0155(3.2e-109)	XP_024367482.1(5e-06)	ND	extracted axonemepoorly conserved
**FAP51**Cre02.g077600	1939(208)	CC	0030s0082(0.0)	XP_023438985.1(1.9)	ND	extracted axonemepoorly conserved
**FAP327**Cre01.g040600	1794(185.9)	CC	0001s1006(2.2e-154)	XP_024363033.1(0.022)	ND	extracted axonemepoorly conserved
**Cre12.g555378**	959(96)	CC	0007s0026(1e-120)	**STPG2L**XP_024367883.1(0.004)	ND	sperm tail PG repeatpoorly conserved
**Cre03.g181100**	897(93.5)	Low complexity	XP_002946715.1(1e-152)	ND	ND	fraction unknownpoorly conserved
**Cre16.g682850**	864(87)	CC, low complexity	0018s0102(1e-68)	ND	ND	fraction unknownpoorly conserved
**FAP258**Cre07.g325751	754(74)	CC	0003s0163(4.8e-74)	ND	ND	extracted axonemepoorly conserved
**FAP423/MOT12**Cre16.g681301	470(51.6)	IQ, P-loop	0022s015(11e-102)	ND	**C11orf65/MF1**NP_689800.3(3e-32)	fraction unknownrelated to conserved protein found in testis
**FAP271**Cre12.g486700	384(40)	Low complexity	0016s0163(4.3e-141)	**STPG2L**XP_024367883.1(0.052)	**STPG1**EAW95125.1(2e-4)	sperm tail PG repeat, weakly related to protein found in testis

Increased in short axonemes, poorly conserved						
**FAP308**Cre10.g430700	3085(315)	CC	0005s0270(9e-118)	ND	ND	extracted axoneme
**CPY**Cre12.g483800	2528(259)	CC, low complexity	0031s0129(1.5e-55)	XP_024392807.1(3.9)	ND	Carboxypeptidasereduced in *pf12; fap20*
**Cre05.g234640**	3529(347)	IQ, low complexity	008s0412(4.2e-51)	ND	ND	extracted axoneme

The proteins and *Chlamydomonas* genome ID numbers are listed in the first column. The total number of amino acids and predicted molecular weight (in parenthesis) are noted in the second column. The predicted protein domains were identified by the SMART program: AAA (ATPase), CC (coiled-coil), EF-hand (calcium binding motif), FN3 (fibronectin-like domain), IQ (calmodulin binding domain), Kelch (beta strand-propeller domain), WD (WD repeat), WW (WW repeat). The predicted amino sequences were compared against the *Volvox carteri*, *Physcomitrella patens*, and the human (*Homo sapiens*) genomes; the names (in bold) and accession numbers of the best hits are shown, along with the BlastP or PHI-Blast score (in parenthesis). ND, not detected or not determined. The proteins reduced in *mbo2* were assorted into groups based on changes in their protein ratios in different mutant axonemes, their susceptibility to extraction with 0.6 M KCl (Pazour *et al.*, 2005), and their relative abundance in WT axonemes (Sakato-Antoku and King, 2022; see Supplemental Table S4).

One unexpected finding was the reduction of DHC5 in *mbo2* axonemes. DHC5 is one of 12 DHCs forming the inner dynein arms (IDA) in *Chlamydomonas* (reviewed in King *et al.*, 2023). To confirm the decrease of DHC5, we probed Western blots of WT and mutant axonemes with DHC5 and DHC9 specific antibodies. DHC5 was reduced in *mbo2* and restored to WT levels in *MBO2-HA* rescued axonemes, whereas DHC9 was present at WT levels in all samples (Figure 2C). Proteomic analysis indicated no significant changes in any of the other DHCs in *mbo2* axonemes compared with WT (Supplemental Table S4). Although the mutant gene products of *mbo1* and *mbo3* remain uncharacterized, both MBO2 and DHC5 were reduced in *mbo1* and *mbo3* axonemes (Figure 2D; Supplemental Table S4). These observations suggest that MBO2 and its associated proteins may stabilize assembly of DHC5 onto the DMT. To better understand the relationship between DHC5 and MBO2, we tested whether the two polypeptides might cofractionate following biochemical extraction of purified axonemes. The IDAs can be extracted from WT axonemes by treatment with 0.6 M NaCl or KCl (Pazour *et al.*, 1995), but extraction of MBO2 required treatment with 0.4–0.6 M NaI or 0.5% Sarkosyl (Figure 2, E and F), indicating that they are in distinct biochemical complexes. A subset of the proteins reduced in *mbo2* are enriched in 0.6M NaCl/0.6M KCl extracts of WT axonemes (Table 3; Supplemental Table S4; Pazour *et al.*, 2005), suggesting that they might be associated with DHC5. The IDAs in 0.6 M NaCl extracts can be partially fractionated by ion exchange FPLC based chromatography into seven distinct peaks containing different dynein isoforms known as IDA *a-g* (Kagami and Kamiya, 1992). DHC5 is the HC subunit of IDA *b* (Yagi *et al.*, 2009). Proteomic analysis of the FPLC peak containing IDA *b* and DHC5 identified several polypeptides, but only one protein, Cre14.g618300, was also reduced in *mbo2* (Table 2; Supplemental Figure S2B; Supplemental Table S4). These results indicate that most of the MBO2-associated proteins dissociate from DHC5 during high salt extraction and FPLC fractionation and therefore do not qualify as *bona fide* subunits of the IDA *b* complex. However, at least some of proteins reduced in *mbo2* may stabilize binding of IDA *b* to the DMT, similar to the way that subunits of the N-DRC stabilize the binding of IDA *e* and *g* (Bower *et al.*, 2013).

### Comparative proteomics of other mutants with phenotypes similar to mbo2

The *pf12/pacrg and pf12; fap20* mutants swim forwards with a symmetric waveform, lack beaks inside the B-tubules of DMTs 5 and 6, and assemble reduced levels of IDA *b* into the axoneme (McVittie, 1972; Frey *et al.*, 1997; Dymek *et al.*, 2019, see Table 1). Given these similarities in phenotypes with *mbo* strains, we analyzed the proteomes of *pf12* and *pf12; fap20* axonemes to look for potential overlap with the proteins missing in *mbo1* and *mbo2*. The *pf12* mutation is a null allele of the *PACRG* gene, which encodes one of two proteins that form the inner junction between the A- and B-tubules of the DMT (Dymek *et al.*, 2019; Figure 1). FAP20 is the second subunit of the inner DMT junction (Yanagisawa *et al.*, 2014). Proteomics of *pf12* axonemes (using iTRAQ labeling) and *pf12; fap20* axonemes (using TMT labeling) confirmed that PACRG is reduced to < 10% of WT levels, whereas MBO2 is present at WT levels, as also shown by Western blot (Supplemental Table S4; Figure 2G; Dymek *et al.*, 2019). However, both *pf12* and *pf12; fap20* showed significant reductions in other axonemal proteins, including several proteins that were reduced in *mbo2* (Tables 1–3; Supplemental Table S4). The shared proteins may include potential docking factors for IDA *b*/DHC5, beak components, or subunits of other complexes destabilized in *mbo, pacrg*, and *fap20* mutants (Tables 1–3).

The *mbo* mutants also have defects in assembly of a subset of proteins recently associated with the FAP57 complex (Tables 1–3; Supplemental Table S4; Lin *et al.*, 2019). FAP57 is a conserved, WD repeat, coiled-coil protein that extends from the MIA complex to beyond the distal end of the 96-nm repeat (Figure 1). Null mutations in *FAP57* (*bop2, ida8, fap57*) have a slow swimming phenotype, partially suppress the symmetric waveforms of *pf10* mutants, and disrupt assembly of FAP57, an EF-hand, WD repeat protein known as FAP337, and three IDA HCs (Dutcher *et al.*, 1988; Lin *et al.*, 2019; Bustamante-Marin *et al.*, 2020; Table 1; Supplemental Table 4). The loss of FAP57 in these mutants is partially offset by increases in the assembly of two paralogues, FBB7 and FAP331. Likewise, the decrease in FAP337 is partially offset by increases in its paralogue, Cre07.g313830 (Lin *et al.*, 2019; Bustamante-Marin *et al.*, 2020; Table 1; Supplemental Table 4). We, therefore, compared the proteomes of *mbo2*, *pf12*, *pf12*; *fap20*, and *ida8* axonemes to determine the extent of overlap between the different polypeptide defects.

As described in Supplemental Table 4, the FAP57 paralogue FAP331 was reduced in axonemes from *mbo*, *pf12,* and *pf12; fap20* strains, FAP57 levels were WT in all mutants, and FBB7 was variably reduced in *pf12* and *pf12; fap20*. Likewise, the two EF-hand, WD repeat proteins, FAP337 and Cre07.g313850, were reduced with variable degrees of significance in the different mutants (Supplemental Table S4; Table 3). However, none of the other polypeptides reduced in *mbo2* were significantly altered in the *ida8/fap57* axonemes (Supplemental Table S4). These results suggest that fluctuations in assembly of the FAP57 paralogues and their associated subunits are secondary defects due to the absence of other proteins in *mbo*, *pf12*, and *pf12; fap20* axonemes (see *Discussion*).

We recently analyzed the proteome of a *pf9-2; pf28* double mutant strain with short flagella (∼2.9 μm) using iTRAQ labeling and MS/MS to identify proteins that might be enriched in the proximal or distal regions of the axoneme (Hwang *et al.*, 2024). DHC5 is reduced in the proximal 2 μm of WT flagella and reduced in short flagella during regeneration (Yagi *et al.*, 2009). Proteomic analysis of short *pf9-2; pf28* axonemes confirmed that the proximal DHCs (DHC3, DHC4, DHC11) were increased > twofold (see Table 2 in Hwang *et al.*, 2024). Here, we examine the levels of the MBO2-associated proteins and confirmed that DHC5 was significantly reduced (∼0.29) in short axonemes relative to WT axonemes (Table 3; Supplemental Table S4). Interestingly, the MBO2-associated proteins FAP324, FAP343, and FAP238, were also reduced below 0.75 in short axonemes (Supplemental Table S4); these proteins were also found in high salt extracts and may potentially interact with DHC5 (Table 3). Three MBO2-associated proteins were increased in *pf9-2; pf28* axonemes (mutant/WT ratio > 1.4), and all were poorly conserved outside green algae (Supplemental Table S4; Table 3). These proteins may be associated with structures located in the proximal region. However, most MBO2-associated proteins were found at WT levels in *pf9-2; pf28* axonemes (Supplemental Table S4). Most FAP57-associated proteins were found at WT levels in short flagella, but both FAP331 and FAP337 were reduced (Supplemental Table S4). Thus, most MBO2-associated and FAP57-associated proteins are found along the length of the axoneme, but a subset may vary between the proximal and distal axoneme.

### Characterization of other mutants with defects in DHC5/IDA b

Given the decrease in DHC5 observed in *mbo* axonemes, we analyzed other mutants with DHC5 defects to determine if the loss of DHC5 might reduce swimming velocity, alter the ciliary waveform, or change swimming direction. The *IO* mutant (CC-2012) is an isolate of *pf23* that assembles full-length, paralyzed flagella lacking most of the IDAs, including IDA *b*/DHC5. However, Western blots showed that MBO2 levels were WT (Figure 2G). We also searched the CLiP library of insertional mutants (Li *et al.*, 2016; 2019) to identify potential *dhc5* mutations (Supplemental Table S1). PCR of genomic DNA identified four strains with plasmid insertions into exons located in the 5′ region of the *DHC5* gene (Supplemental Figure S3, A–C). Western blots of axonemes probed with a DHC5 antibody detected bands migrating at the sizes predicted for N-terminal fragments containing the tail domain but lacking the C-terminal motor domain. MBO2 was also present at WT levels (Supplemental Figure S3D). Analysis of swimming phenotypes showed that the *dhc5* strains swam forwards at WT speeds with no obvious motility defects (Supplemental Figure S3E), demonstrating that absence of the DHC5 motor domain does not result in a *mbo* phenotype.

### Cryo-electron tomography reveals defects in assembly of IDA b and several DMT-specific structures

**FIGURE 3:**
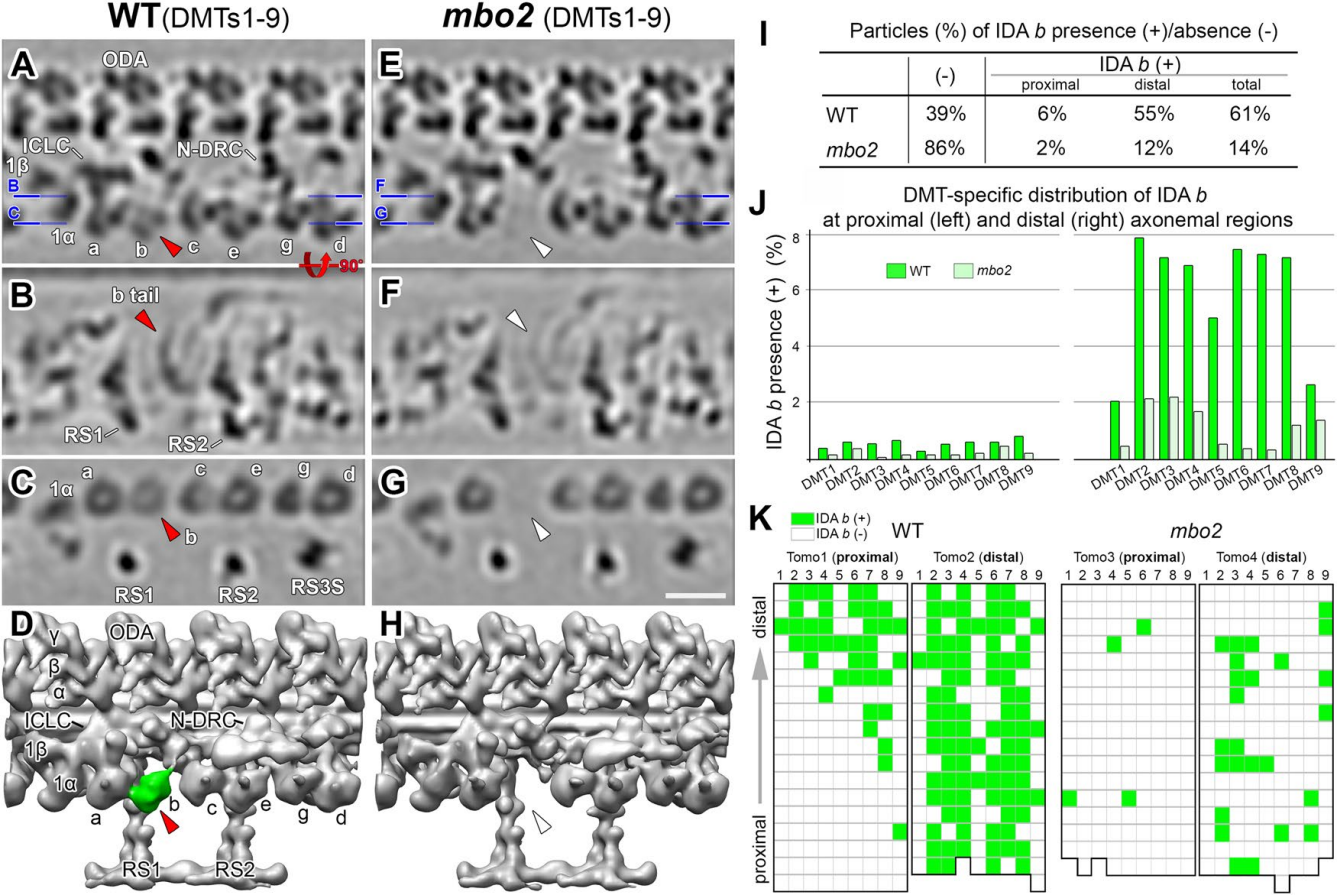
IDA *b* is reduced in the 96 nm axonemal repeat from *mbo2*. (A–H) Tomographic slices (A–C, E–G) and isosurface renderings (D and H) of the averaged 96-nm repeats from all DMTs of WT (A–D) and *mbo2* (E–H) axonemes. Blue lines in (A and E) indicate the locations of the slices in the respective panels. IDA *b* can be visualized in WT (red arrowheads) but not in *mbo2* (white arrowheads). (I) The presence (+) and absence (–) of IDA *b* in the 96-nm repeats from the proximal and medial-distal regions of WT and *mbo2* axonemes. (J) The bar graph shows the fraction of repeats with IDA *b* for each DMT from WT (green) and *mbo2* (light green) in the proximal and distal regions. (K) Distribution pattern of IDA *b* in four tomograms of proximal and distal regions from WT (left) and *mbo2* (right). Each grid represents a single repeat, and the colors indicate whether IDA *b* is present (green) or absent (white) in the repeat. From bottom to top, the grids represent the repeats in the proximal-to-distal direction (gray arrow). Note that IDA *b* is not present in the proximal region and Tomo 1 of WT shows a transition from the proximal-to-distal region. Other labels: 1α, β, the I1 dynein α- and β-head; a-g, single-headed IDAs; ICLC, intermediate chain, and light chain complex of I1 dynein; IDA, inner dynein arm; N-DRC; ODA, outer dynein arm; RS1/2/3S, radial spokes one, two, and three stand-in. Scale bar in G, 20 nm (valid for A–C and E–G).

Studies of *mbo* axonemes by conventional TEM have shown defects in the assembly of beak structures within the lumens of the B-tubules of DMT5 and DMT6 but failed to reveal any defects in the assembly of the IDAs (Segal *et al.*, 1984; Tam and Lefebvre, 2002). These earlier studies analyzed random cross-sections of fixed axonemes and did not employ any image averaging. We used cryo-ET, computational averaging of the 96-nm repeat, and classification analysis to reanalyze various structures, including the IDAs, in WT and *mbo2* axonemes (Figure 3). Sub-tomogram averages of 96-nm repeats from all WT DMTs revealed the two-headed I1/*f* dynein at the proximal end of the repeat, followed by the six, single-headed IDAs (*a, b, c, e, g, d*; Figure 3, A–D). However, the electron density of IDA *b*, which contains DHC5 and is located just distal to RS1, was weaker than for other IDAs, suggesting IDA *b* was not present in all WT 96-nm repeats (Figure 3, A–D). In contrast to WT, tomograms of the 96-nm repeat from all DMTs in *mbo2* lack IDA *b* almost entirely (Figure 3, E–H). As previously reported for WT axonemes, IDA *b* is significantly reduced on all DMTs in the proximal region of the axoneme (Yagi *et al.*, 2009) and specifically reduced on certain DMTs in the medial/distal region (Bui *et al.*, 2012; Lin *et al.*, 2012, 2019; Dymek *et al.*, 2019). Therefore, we performed classification analyses to determine the presence or absence of IDA *b* in different regions of the axoneme and on specific DMTs (Figure 3, I and J). In WT, IDA *b* was present in 6% of the proximal tomograms and 55% of the medial/distal tomograms; in *mbo2*, IDA *b* was only present in 2% of the proximal tomograms and 12% of the medial/distal tomograms (Figure 3I). In WT axonemes, IDA *b* was reduced on all DMTs in the proximal region and on DMT1, DMT5, and DMT9 in the medial/distal region (Figure 3J), consistent with previous reports (Bui *et al.*, 2012, Lin *et al.*, 2012, 2019; Dymek *et al.*, 2019). In *mbo2*, IDA *b* was reduced in all DMTs, with the most significant changes from WT in the medial/distal region of DMT2 to DMT8 (Figure 3J). Examples of the distribution of IDA *b* in individual axonemes are shown in Figure 3K.

**FIGURE 4:**
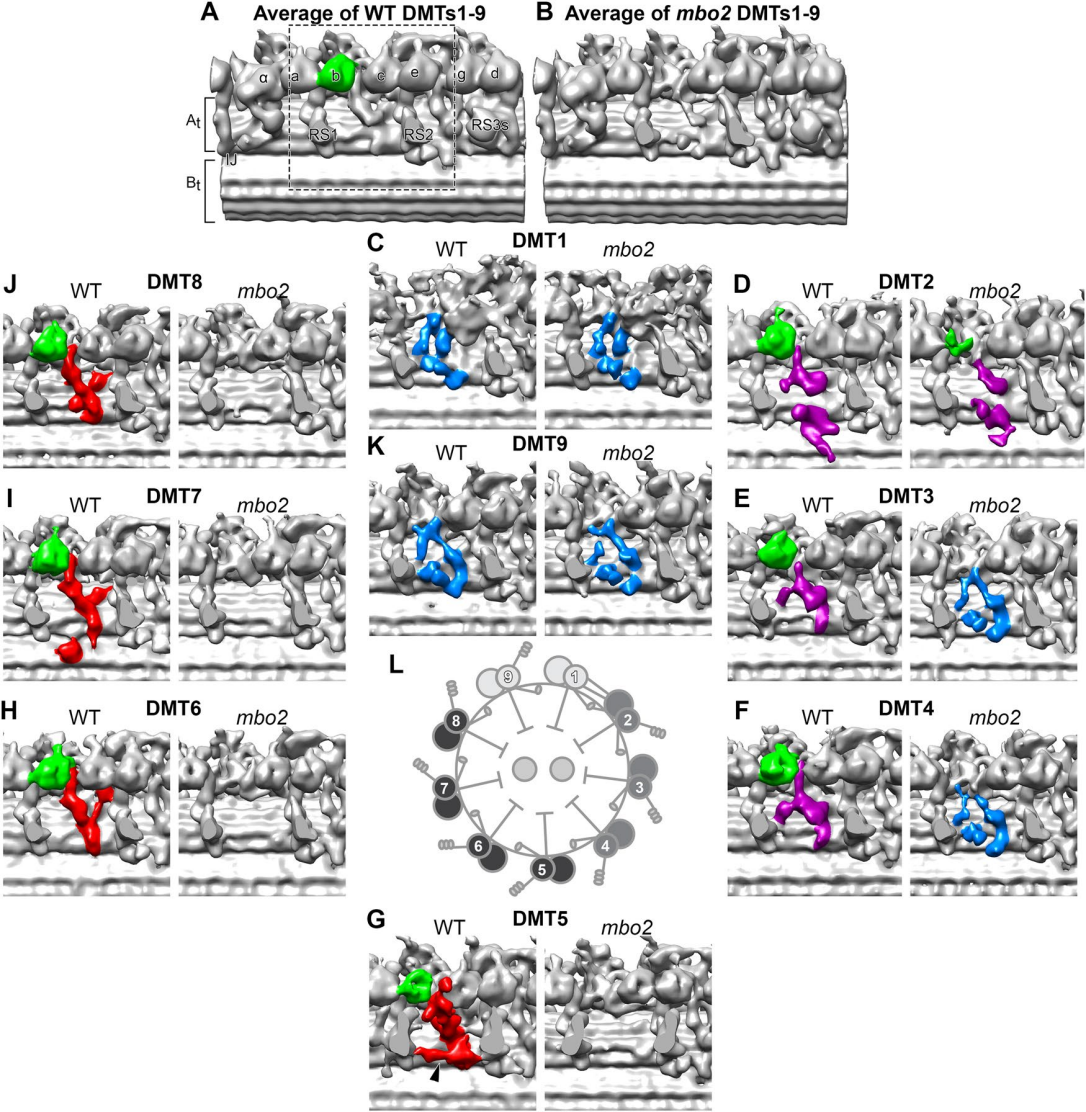
DMT specific defects in *mbo2*. (A and B) Bottom view of the three-dimensional isosurface renderings of the averaged 96-nm repeats from all DMTs in WT (A) and *mbo2* (B) axonemes. Radial spokes (RS) were cropped to allow an unhindered view of the IDA-to-DMT-docking region. Motor domain of IDA b is colored green (C–K) Comparison of the IDA *b* tail-associated structures across nine DMTs between WT (left) and *mbo2* (right) focusing on the region marked by the dashed box in A. Based on the structural characterizations in WT and defects in *mbo2*, the WT DMTs were categorized into three groups: DMTs1, nine (blue), DMTs 2–4 (purple) and DMTs 5–8 (red). The black arrowhead in WT DMT5 indicates a DMT5-specific structure connecting RS1 and RS2. (L) A schematic drawing of the axoneme in cross-section to show the asymmetric distribution of the IDA *b* associated structures on the three classes of DMTs.

Because several polypeptides are missing or reduced in *mbo2* axonemes, we analyzed the region around IDA *b* using DMT-specific averaging (Figure 4). The averages of DMT1 and DMT9 did not show significant changes between WT and *mbo2* (Figure 4, C and K). These results are consistent with the observation that IDA *b* was rarely found on these DMTs in either WT or *mbo2* (Figure 3J). For DMT5 to DMT8, both IDA *b* and the WT structures near the base of IDA *b* were completely missing in *mbo2* (Figure 4, G–J). This suggests that a subset of the proteins missing in *mbo2* are located within the structures around the base of IDA *b*. In contrast, the *mbo2* defects were different in the averages of DMT2 to DMT4. Even though IDA *b* was missing in *mbo2*, the region surrounding the base of IDA *b* retained significant structural density, similar but not identical to the structures seen in WT (compare purple/blue density in Figure 4, D–F). These structures may contain other proteins that are related to proteins missing in *mbo2*, but that do not depend on MBO2 for assembly into the axoneme (see *Discussion*).

**FIGURE 5:**
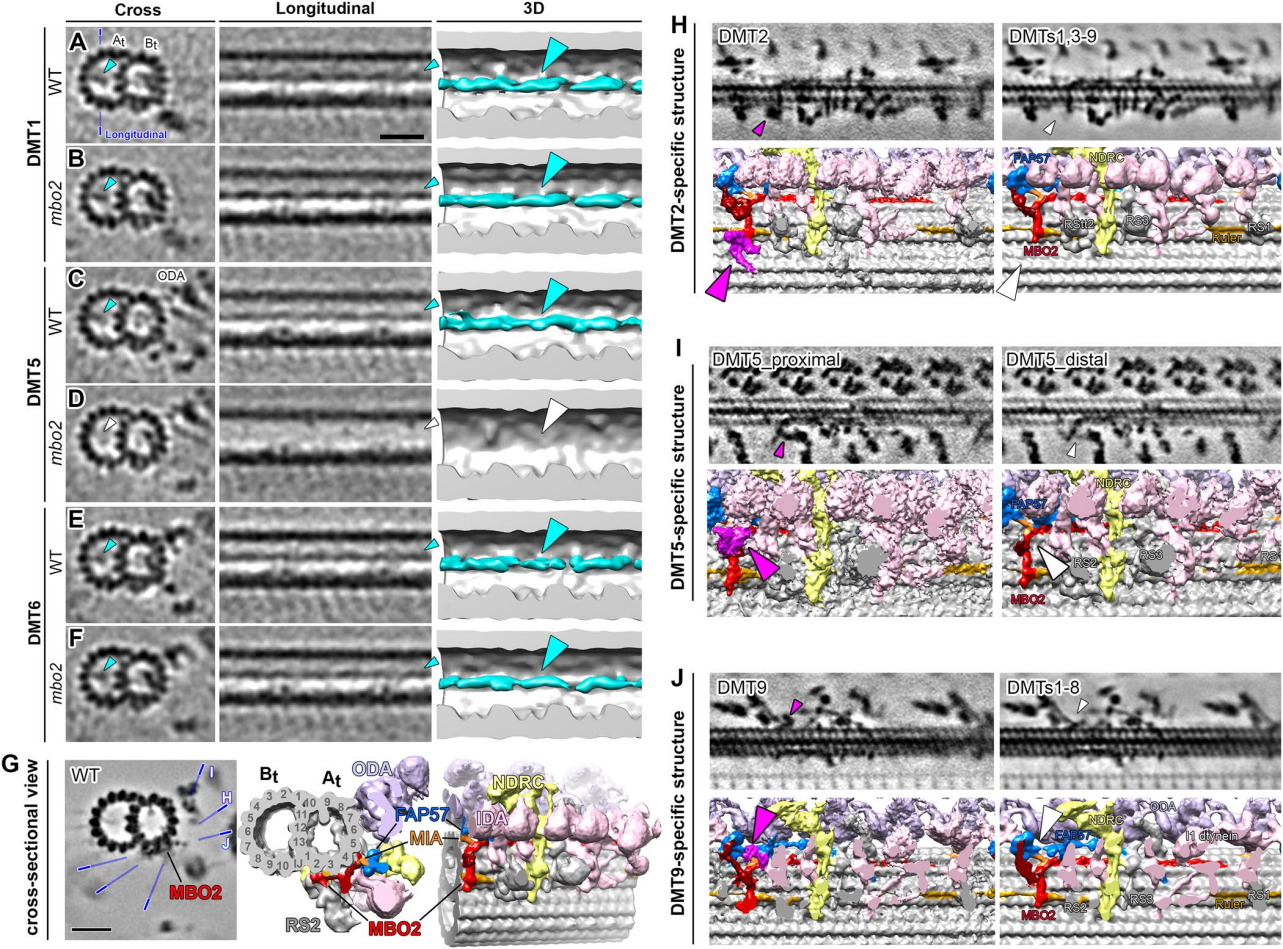
The ciliary axoneme contains several DMT specific structures. (A–F) The B-tubule beak structures are found in DMTs 1, 5, and 6 of WT axonemes but missing in DMT5 of *mbo2* axonemes. Tomographic slices (left two columns viewed in cross-sectional and longitudinal orientations) and three-dimensional isosurface renderings (viewed in longitudinal orientation) show the B-tubule beak structure in DMT1 (A and B), DMT5 (C and D), and DMT6 (E and F) of WT and *mbo2* axonemes. The blue line in (A) indicates the location of the longitudinal slices. Cyan arrowheads indicate presence of the beak structure, which is missing in DMT5 in *mbo2* (D, white arrowheads). (G) Tomographic slice (left) and three-dimensional isosurface renderings (right) of the averaged WT DMT structure viewed from cross-sectional (the left two columns) and bottom-up (the 3^rd^ column) orientations. The density corresponding to the MBO2 protein and its associated MIA, FAP57 protein are indicated. (H–J) Tomographic slices (top row) and three-dimensional isosurface renderings (bottom row) showing three representative DMT specific structures that are located near the L-shaped MBO2 filament. Note that the orientations of the tomographic slices are indicated by the blue lines in (G). In (H), a density extending from the C-terminal region of MBO2 was only observed on DMT2 (magenta arrowheads) but not on other DMTs (white arrowheads). In (I), a structure that attaches near the base of the IDA *b* tail domain was identified only in the proximal region of DMT5 (magenta arrowheads). This density was not seen in the distal region of DMT5 (white arrowheads) or on other DMTs. In (J), an extra density protruding from the FAP57 complex was only found on DMT9 (magenta arrowheads) but not on other DMTs (white arrowheads). Scale bars in (A) and (G) are 20 nm and valid for the EM images in (A–F) and (G–J), respectively.

Tomograms from the proximal region of the axoneme were also analyzed for the presence or absence of the beak structures inside the B-tubules of DMT1, DMT5, and DMT6 (Figure 5). Beak structures were visible in DMT1 and DMT6 from both WT and *mbo2* axonemes (Figure 5, A, B, E, and F) but not visible in DMT5 from *mbo2* axonemes (Supplemental Figure 5, C and D). DMT-specific averaging also revealed several other densities associated with the MBO2 complex that varied between DMTs in WT axonemes; several examples are shown in Figure 5, G–J.

### Rescue of mbo2 defects and mapping the position of MBO2 by transformation with SNAP-tagged MBO2 transgenes

**FIGURE 6:**
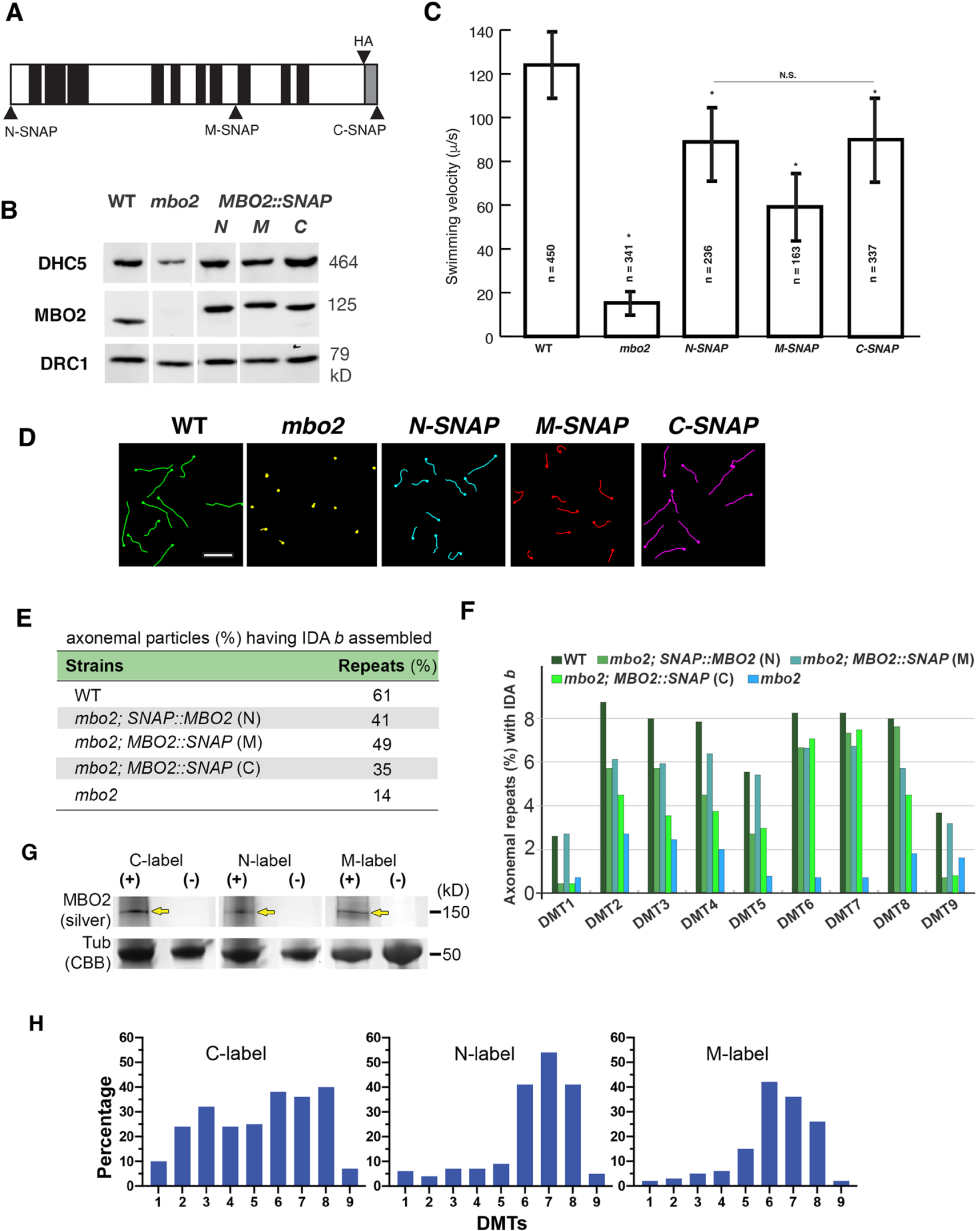
Rescue of biochemical, motility, and structural defects in the SNAP-tagged *MBO2* strains. (A) Schematic diagram of MBO2 showing the insertion of SNAP tags at the N-terminus (N-SNAP, M1), in the middle (M-SNAP, L569) and at the C-terminus (C-SNAP, I920) of the polypeptide. Also shown are the coiled-coil domains (black), disordered region (gray) and HA tag. (B) Western blot of axonemes from WT, *mbo2*, and *MBO2::SNAP* rescued strains (N, M, and C) probed with different antibodies. Both DHC5 and MBO2 are reassembled in the rescued strains; DRC1 is a loading control. (C) Transformation of *mbo2* with each construct significantly increased (*P* < 0.05) swimming velocity relative to *mbo2* (see asterisks). *N-SNAP* and *C-SNAP* were significantly faster than *M-SNAP*, slower than WT, but not significantly different from one another. (D) Forward swimming trajectories of each strain are shown here for an interval of 1 s. (The *mbo2* strain is swimming backwards.) Scale bar, 50 mm. (E) Percentage of 96-nm axoneme repeats that contain IDA *b* in WT, *mbo2*, *mbo2; MBO2::SNAP* (N), *mbo2; SNAP::MBO2* (M) and *mbo2; MBO2::SNAP* (C) strains. The assembly of IDA *b* is increased in the rescued strains as compared with *mbo2*. (F) Bar graph shows the percentage of 96-nm repeats that contain IDA *b* for each DMT from WT, the three SNAP-tagged rescued strains, and *mb*o*2*. IDA *b* is increased on DMTs 2–8 in all rescued strains as compared with *mbo2*. (G) SDS–PAGE of SNAP-tagged axonemes labeled with streptavidin gold and detected by silver enhancement. The yellow arrows mark the SNAP-tagged MBO2 polypeptides. Tubulin was stained with Coomassie Brilliant Blue (CBB) as a loading control. (H) Percentage of 96-nm repeats with gold-labeled SNAP-tags found on each DMT in *mbo2; MBO2::SNAP* (C), *mbo2; SNAP::MBO2* (N) and *mbo2; MBO2::SNAP* (M).

The large number of MBO2-associated proteins and the complexity of structural defects in *mbo2* axonemes make precise localization of the MBO2 subunit impossible using a WT-mutant comparison. Therefore, we constructed three *MBO2* transgenes with SNAP tags located at the N-terminus (N-SNAP), near the middle of the protein (M-SNAP at amino acid 569) and at the C-terminus (C-SNAP; Figure 6A; Supplemental Figure S1). Each transgene was introduced into *mbo2* by cotransformation, and transformants were screened for recovery of forward swimming and reassembly of MBO2 and DHC5 into the axoneme. Western blots of axonemes from rescued strains demonstrated that MBO2 was assembled at WT levels and migrated at the size expected for a SNAP-tagged subunit. Assembly of DHC5 was also restored in the rescued strains (Figure 6B). Measurement of swimming velocities indicated the N-SNAP and C-SNAP rescued strains recovered ∼72% of WT velocity, whereas the M-SNAP rescued strain recovered only ∼48% of the WT speed (Figure 6C). However, analysis of cell trajectories and motility by phase contrast microscopy confirmed that all the rescued strains were swimming forwards with asymmetric waveforms (Figure 6D). The SNAP-tagged transgenes therefore restored sufficient function to rescue the *mbo* phenotype, even though the rescued cells were not completely WT with respect to swimming velocities.

To obtain a more quantitative measurement of recovery, we analyzed the DHC composition of the rescued strains by SDS–PAGE, mass spectrometry, and cryo-ET. Axonemes from WT, *mbo2-4*, and the three SNAP-tagged rescued strains were fractionated by SDS–PAGE, and the DHC region was excised for MS/MS analysis (see boxed region in Supplemental Figure S2C). The relative abundance of each IDA DHC was estimated by spectral counting and plotted as a percentage of DHC content of the I1 dynein (Supplemental Figure S2D). Quantification of peptides using the tools available in Proteome Discover yielded similar results (Supplemental Table S4). Only DHC5 was significantly reduced in *mbo2*, and it was reassembled in the SNAP-tagged rescued axonemes at 64–84% of WT levels. Inspection of tomograms from the medial and distal regions of axonemes confirmed that IDA *b* was present in 14% of the 96-nm repeats in *mbo2* and increased to 35–49% of the repeats in the SNAP-tagged rescued strains, compared with 61% of the repeats in WT (Figure 6E). Analysis of individual DMTs showed that recovery of IDA *b* in the SNAP-tagged strains could be observed on DMT2 to DMT8, with the C-SNAP closest to WT and M-SNAP showing more DMT-specific variability, reaching almost WT levels on DMTs 6 and 7, but significantly less on the remaining DMTs (Figure 6F).

**FIGURE 7:**
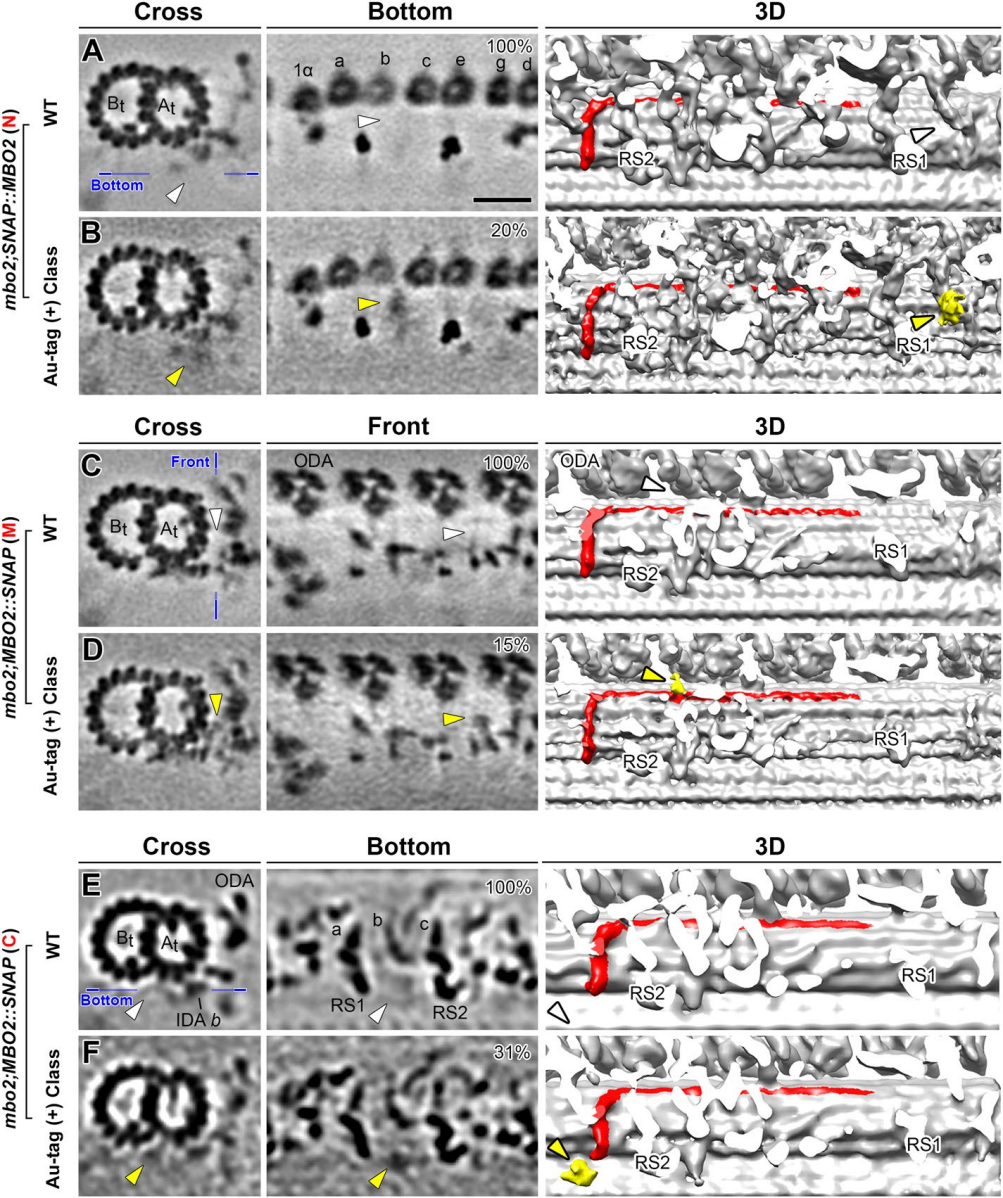
Localization of the N-terminus, middle region, and C-terminus of the MBO2 polypeptide in the 96-nm axoneme repeat. The locations of the three different SNAP tags were revealed by comparing WT (A, C, and E) with streptavidin-gold labeling of different SNAP-tagged rescued strains (B, D, and F) and class averaging of the axonemal repeats with tag-density. Shown are tomographic slices (left two columns) and three-dimensional isosurface renderings (third column). (A and B) The region containing the structures associated with the tail domain of IDA *b* are viewed in cross-sectional and bottom orientations in WT (A) and *mbo2; N-SNAP::MBO2* rescued axonemes (B). The blue line in (A) indicates the location of the slices viewed in the bottom orientations. The L-shaped, MBO2-associated density of one 96-nm repeat is colored in red in the three-dimensional isosurface renderings (A–F). Classification analysis revealed an additional density near the tail domain of IDA *b* in ∼20% of the repeats (indicated by yellow arrowheads in B). WT repeats lack similar density in this position (white arrowheads in A). (C and D) Tomographic slices viewed in cross-sectional and front orientations and cropped three-dimensional isosurface renderings viewed in front orientation of the averaged repeats from WT (C) and *mbo2; MBO2::M-SNAP* rescued axonemes (D). The blue line in (C) indicates the location of the slice viewed in front orientation. Classification analysis of *mbo2; MBO2::SNAP* (M) averages revealed an additional density (yellow arrowheads in D) in ∼15% of the repeats; this density is located on the surface of the A-tubule between protofilaments A04 and A05. A similar density in this was not observed by classification of the WT repeats (white arrowhead in C). (E and F) Tomographic slices and three-dimensional isosurface renderings viewed in cross and bottom orientations from WT (E) and *mbo2; MBO2::C-SNAP* (F) axonemes in the region close to the surface of the DMT below IDA *b*. The blue line in (E) indicates the locations of the slices viewed in the bottom orientations in (E) and (F). Classification analysis of the *mbo2; MBO::C-SNAP* rescued axonemes revealed an additional density (yellow arrowheads in F) in ∼31% of the repeats. This density indicates the likely location of the C-terminus of MBO2 close to the inner junction. A similar density was not seen by classification of WT axonemes (white arrowheads in E). Other labels: A_t_/B_t_, A- and B-tubule; a-g, single-headed IDAs; ODA, outer dynein arm; RS, radial spoke. Scale bars in A, C, and E, 20 nm (valid for all EM images).

To determine how the MBO2 polypeptide might be arranged relative to the structures associated with IDA *b*, we treated axonemes with biotin-streptavidin gold (∼1.4 nm) to label the SNAP tags for in situ visualization by cryo-ET. Control experiments using silver enhancement procedures to stain samples on gels (Song *et al.*, 2015) confirmed that the SNAP-tags were accessible to streptavidin-gold (Figure 6G). To identify additional densities, that is, density not visible in WT averages and thus corresponding to the streptavidin-gold labels, we classified the subtomogram volumes of the 96-nm repeats, revealing the tag-density at distinct locations within the repeat (Figures 6H and 7). Specifically, in the N-SNAP rescued strain, the recovery of IDA *b* was evident in 41% of the averaged repeats compared with 61% in WT (Figure 6E), and an additional label-density was observed close to the IDA *b* tail domain in ∼20% of the repeats (compare white and yellow arrowheads in Figure 7, A and B). In the M-SNAP strain, 49% of the repeats contained IDA *b* (Figure 6E), and an additional label-density was observed near the distal end of the MBO2-associated complex in ∼15% of the repeats (yellow in Figure 7D). In the C-SNAP strain, 35% of the repeats contained IDA *b* (Figure 6E), and an additional label-density was observed close to the IDA *b* tail and inner junction region in ∼31% of the repeats (yellow in Figure 7F). This suggests that both the N-terminus and C-terminus of MBO2 are located between RS1 and RS2, in the vicinity of the IDA *b* attachment site, possibly stretching a full 96-nm repeat as a L-shaped structure (see *Discussion*).

We did not detect additional densities for streptavidin gold in all repeats of the SNAP-rescues, even though we observed significant recovery of IDA *b* and MBO2-associated structures in all three SNAP-tagged strains (Figure 6, E and F). Given the differences between DMTs in the *mbo2* mutant, we considered two possibilities. The first is that the accessibility of the SNAP-tags in the MBO2 subunit to the streptavidin-gold particles might vary within the complex of associated proteins in the axoneme. The second is that our ability to detect the SNAP tags might vary between different DMTs. We, therefore, checked the tomograms to determine which DMTs were associated with the label densities (Figure 6H). We found that the C-terminal tag was detected mostly on DMT2 to DMT8. However, the N-terminal and M-SNAP tags were detected more frequently on DMT6 to DMT8 than other DMTs. These observations suggest that the MBO2 subunit is located on DMT2 to DMT8, but the N- and M-SNAP tags are more accessible to gold labeling on DMT6 to DMT8.

## DISCUSSION

### Characteristics of proteins missing or reduced in mbo axonemes and their potential interactions

MBO2 is a conserved, coiled-coil protein that is tightly bound along the length of the axoneme, and two-dimensional-PAGE previously indicated that *mbo* mutants have defects in the assembly of at least eight unidentified axonemal proteins (∼33–245 kD; Segal *et al.*, 1984; Tam and Lefebvre, 2002). We used five rounds of isobaric labeling (iTRAQ or TMT) and quantitative MS/MS to identify multiple proteins that were missing or reduced in *mbo2* axonemes and restored in *MBO2-HA* rescued axonemes (Table 2; Supplemental Table S4). Here we discuss the 24 most abundant polypeptides that were significantly reduced in the TMT experiment as potential candidates to be part of a MBO2 complex (Tables 2 and 3). To gain further insight into these proteins, we analyzed their susceptibility to extraction with high salt (0.6M NaCl/KCl), their cofractionation with IDAs by FPLC, and their relative abundance in other motility mutants that alter waveform asymmetry or ciliary length. The proteins were sorted into five groups whose characteristics are summarized in Table 3.

The first group contains MBO2 and four other proteins, FAP58, Cre06.g289600, Cre14.g618300, and FAP145, that may coassemble with MBO2 as part of the L-shaped structure in the 96-nm axoneme repeat. All four proteins are relatively abundant, coiled-coil proteins, significantly reduced only in *mbo2*, but not altered in *pf12, pf12; fap20*, or short flagella mutants (Tables 1–3; Supplemental Table S4; Pazour *et al.*, 2005; Lin *et al.*, 2019; Sakoto-Antoku and King, 2022). Cre06.g289600 is not highly conserved, but MBO2 and FAP58 share significant sequence homology (33.5% identity, Blast P score 3e-44) and even greater homology to different proteins in other species with motile cilia and flagella (Nevers *et al.*, 2017). FAP58 is also closely related (∼80% identity) to a *Chlamydomonas* paralogue, FAP189. FAP58, however, is reduced in all mutants with a *mbo* phenotype, whereas FAP189 is unchanged or possibly elevated in *mbo* strains (Tables 2 and 3; Supplemental Table S4). Thus, the FAP58 and FAP189 paralogues may play distinct but complementary functions in *Chlamydomonas*. A recent cryo-EM, single particle analysis visualized parts of the L-shaped structure on the A-tubule with higher resolution, revealing a coiled-coil filament. The authors proposed a pseudoatomic model, placing a FAP189 homodimer within the L-shaped coiled-coil structure on the surface of the DMT (Walton *et al.*, 2023). We propose that MBO2 and FAP58 form a coiled-coil heterodimer that complements the role of a FAP189 homodimer on different DMTs or regions. Recent chemical crosslinking and proteomic analysis of the MBO2 and FAP58 orthologs in *Tetrahymena* indicate that these two subunits interact closely throughout their lengths in the *Tetrahymena* axoneme (McCafferty *et al.*, 2023), consistent with our hypothesis. MBO2 is the orthologue of the vertebrate polypeptide CCDC146 (29.5% identity, Blast P score 1e-103), and *ccdc146* mutations result in defective sperm formation and male infertility (Muroňová *et al.*, 2023). Interestingly, there is only a single orthologue of FAP58 in most species (Nevers *et al.*, 2017), known as CFAP58/CCDC147 in vertebrates (∼50% identity). CCDC147 is abundantly expressed in the testis and to a lesser extent in the lung, heart, and endometrium, and *ccdc147* mutations are associated with abnormal sperm flagella morphology and rare germline mutations associated with lung cancer (Liu *et al.*, 2016; He *et al.*, 2020; Sha *et al.*, 2021). Whether *ccdc146* or *ccdc147* mutations alter ciliary waveforms in other species is currently unknown.

Two smaller proteins are also reduced only in *mbo2*, FAP145 and Cre14.g618300, and both can be extracted in high salt (Table 3; Supplemental Table S4) FAP145 is weakly related to a translin associated factor X interacting protein but is otherwise uncharacterized. Cre14.g618300 was detected in FPLC peak *b*, along with DHC5. Additional studies are needed to determine whether these proteins might facilitate interactions between the MBO2/FAP58 heterodimer and IDA *b.*

The second group of proteins reduced in *mbo2* includes two proteins previously associated with the FAP57 complex, FAP331 and FAP337 (Lin *et al.*, 2019; Bustamante-Marin *et al.*, 2020). Both are extracted in high salt and altered in *ida8/fap57* mutants (Supplemental Table S4; Tables 1–3). The FAP57-associated proteins also fluctuate in *mbo1*, *pf12,* and *pf12; fap20* strains. These are consistent with observations that the FAP57 complex interacts with multiple structures in the axoneme, including an L-shaped structure (Lin *et al.*, 2019; Ghanaeian *et al.*, 2023; Walton *et al.*, 2023, see below).

To identify other polypeptides that might facilitate interactions between DHC5 and MBO2, we searched for proteins that were enriched in high salt extracts and significantly reduced in short (*pf28; pf9-2*) axonemes (Supplemental Table S4; Pazour *et al.*, 2005; Hwang *et al.*, 2024). This group contains DHC5 and three other polypeptides, FAP324, FAP238, and FAP343 (Tables 1–3; Supplemental Table S4). FAP324 (∼70 KD) is significantly reduced below 17% in all *mbo* proteomics experiments and contains an N-terminal coiled-coil domain and C-terminal Kelch and FN3 domains. It shares limited homology to KLHDC3, which is abundantly expressed in the testis. FAP238 is a small (42 kD), coiled-coil protein with an EF-hand domain that may function as a calcium sensor. FAP343 is weakly related to CEP164. Both FAP238 and FAP343 are also reduced in the *pf12* strains (Table 3).

A larger group of proteins were reduced in either *pf12* and/or *pf12; fap20* but not altered in short axonemes (Table 3; Supplemental Table 4). Cre3.g144887 is a relatively abundant protein that contains an IQ domain and a LRRC domain with limited homology to the LRRC74a protein found in testis. This protein is also present in salt extracts. FAP423/MOT12 is a minor component based on previous proteomes (Pazour *et al.*, 2005, Sakoto-Antoku and King, 2022), but it contains an IQ motif and is related to a human protein, C11orf65, that is relatively abundant in testis and conserved in other species with motile axonemes (Li *et al.*, 2004). Two other proteins, FAP271 and Cre12.g555378, share limited homology with a sperm-tail PG-repeat domain containing protein also abundant in testis. The remaining six proteins are coiled-coil or low complexity proteins that were reduced in *pf12* and/or *pf12; fap20* but are poorly conserved outside of green algae.

The last group contains three large (259–348 kD), relatively abundant but poorly conserved proteins that were reduced in *mbo2* but increased >1.4 fold in short axonemes (Tables 1–3; Supplemental Table S4). (Pazour *et al.*, 2005; Sakoto-Antoku and King, 2022). In particular, FAP308 was enriched nearly threefold in short axonemes, suggesting a potential function in the proximal region of the axoneme (Hwang *et al.*, 2024).

### Cryo-ET of mbo2 reveals multiple DMT-specific defects

**FIGURE 8:**
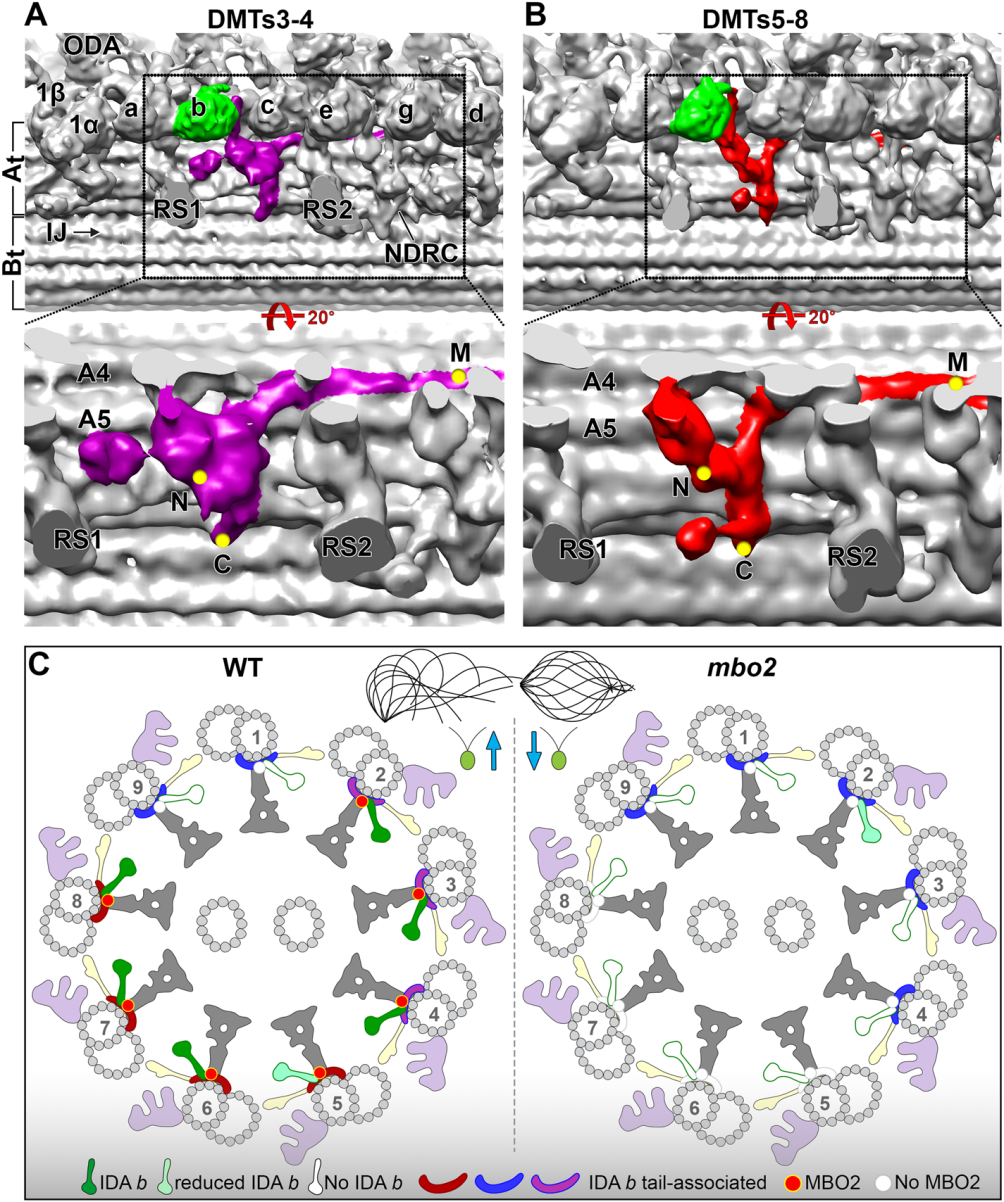
Doublet specific asymmetry of IDA *b* tail-associated structures. (A and B) three-dimensional isosurface renderings of the 96-nm repeats obtained by averaging DMTs 3–4 (A) and DMTs 5–8 (B) of WT axonemes. The regions marked by dashed boxes on the images in the top row were rotated and enlarged in the images shown in the bottom row for better visualization of the structures associated with the IDA *b* tail domain. These structures are highlighted by purple and red colors in DMTs 3–4 and DMTs 5–8, respectively. Locations of the additional densities seen by gold labeling of the C-terminal, N-terminal, and Mid-region SNAP tags in MBO2 are indicated by the yellow dots. (C) Schematic drawings of the cross-section of WT (left) and *mbo2* (right) axonemes showing the arrangement of MBO2 and IDA *b* tail-associated structures across the nine DMTs. Our data suggest that in WT, MBO2 is missing on both DMT1 and nine, because no defects were observed on these DMTs in the *mbo2* mutant. The distinct variations observed in the IDA *b* tail-associated structures on different DMTs are indicated by different colors (green, light green, white). The structure is unchanged between WT and *mbo2* on DMT1 or DMT9 (see blue density) and only slightly affected on DMT2 (purple to magenta). Loss of MBO2 causes the IDA *b* tail-associated structures to be completely missing on DMT5 - DMT8 (white densities) and alters their morphology on DMT3 - DMT4 (from purple to blue), resembling the structures seen on DMT1 and DMT9. Other labels: At/Bt, A- and B-tubule; a-g, single-headed IDAs; 1α, 1β, the I1 dynein α- and β-head; IJ, inner junction; N-DRC; ODA, outer dynein arm; RS1, RS2, radial spoke 1 and 2.

Cryo-ET of *mbo2* axonemes confirmed the loss of IDA *b* in the 96-nm repeat (Figure 3), and transformation of *mbo2* with tagged *MBO2* constructs rescued the *mbo* motility phenotype and restored assembly of DHC5 and IDA *b* into the axoneme (Figures 2 and 6). Thus, although MBO2 and DHC5 are members of distinct biochemical subcomplexes, MBO2-associated proteins are required for stabilizing the assembly of DHC5 at a specific site in the 96-nm repeat. To investigate how these complexes might interact within the axoneme, we turned to DMT-specific averaging. Immunofluorescence microscopy first demonstrated that DHC5 is severely reduced in the proximal 2 μm of WT axonemes (Yagi *et al.*, 2009), and cryo-ET later showed that IDA *b* is also missing or reduced on DMT1, DMT9, and DMT5 in the medial-distal region of WT axonemes (Bui *et al.*, 2012; Lin *et al.*, 2012; 2019; Dymek *et al.*, 2019). In *mbo2*, IDA *b* is reduced on all DMTs (Figures 3 and 4) but reassembled on DMT2 to DMT8 in *MBO2* rescued strains (Figure 6). DMT-specific averaging further revealed that the L-shaped structures located below the tail domain of IDA *b* are heterogenous in appearance on different DMTs in both WT and *mbo2* (Figure 4). More specifically, the L-shaped structure (indicated in red in Figures 4 and 8), located between RS1 and RS2 on DMTs 5 to 8 in WT axonemes is missing on DMTs 5 to 8 in *mbo2*. A slightly different L-shaped structure is present on DMTs 2 to 4 in both strains (indicated in purple in Figures 4 and 8). The heterogeneity in structures suggests that some of the proteins reduced in *mbo2* are located on DMTs 2 to 8, whereas other subunits may be restricted to specific regions of the axoneme or a smaller subset of DMTs.

The *mbo*, *pacrg*, and *fap20* mutants share defects in the assembly of the B-tubule beak-MIP structures located inside DMTs in the proximal region of the axoneme (Segal *et al.*, 1984; Yanagisawa *et al.*, 2014; Dymek *et al.*, 2019). More specifically, *mbo2* lacks beaks in DMT5 (Figure 5), and both *pacrg* and *fap20* display beak defects in DMTs 1, 5, and 6 (Yanagisawa *et al.*, 2014; Dymek *et al.* 2019). How defects at the IJ of the DMT (in the case of *fap20* and *pf12*) or on the surface of the A-tubule (in the case of *mbo2*) are both associated with defects in beak structures in the proximal B-tubules and defects in assembly of IDA *b* along the length of the axoneme is not yet understood. Holes in the IJ of the DMT may alter the stability of MIPs inside the B-tubule and influence the binding of other proteins on the DMT surface. Likewise, some of the proteins missing in the *mbo* strains may interact directly or indirectly with the DMT junction or MIP proteins. Further characterization of mutations in those proteins that are reduced in both the IJ and *mbo* mutants (Table 3; Supplemental Table 4) could provide new insights into the overlapping protein network and the phenotypic similarities between the strains (i.e., symmetric waveforms, defective beaks, reduced IDA *b*). Likewise, further characterization of proteins that are uniquely missing in *mbo* mutants (Table 3) could provide a better understanding of the mechanism that converts the waveform from forwards to backwards swimming (Table 3).

### Epitope-tagged MBO2 constructs rescue the mbo phenotype and most of the DHC5/IDA b defects

Transformation with tagged *MBO2* constructs restores forward swimming, but none of the rescued strains swim at completely WT velocities (Figures 2 and 6; Tam and Lefebvre, 2002). To better understand why swimming velocities might be reduced, we analyzed the reassembly of DHC5/IDA *b* using multiple proteomic approaches and cryo-ET. In general, the recovery of the swimming velocity could be correlated with the extent of DHC5/IDA *b* recovery (Figure 6; Supplemental Figure S2; Supplemental Table S4). However, disruption of the DHC5 motor domain by itself had negligible effects on motility (Supplemental Figure S3). These observations suggest that the interaction of IDA *b* with the MBO2-associated subunits is more critical for regulating motility than the activity of the DHC5 motor domain alone. Interactions within the MBO2 complex may influence coordination with other dyneins in the axoneme and/or alter mechanical properties such as resistance or elasticity. Indeed, interactions between dynein motor and tail domains has been shown to alter motor activity in many systems (McKenney, 2019; Rao *et al.*, 2021).

### Localization of the MBO2 SNAP tags within the 96-nm repeat

Given the heterogeneity of structural defects in the *mbo2* mutant, we turned to streptavidin gold labeling of SNAP tags, DMT-specific averaging, and class averaging to determine the approximate location of the MBO2 polypeptide. Labeling of the C-terminal SNAP tag and DMT-specific averaging suggested that MBO2 is present on DMT2 – DMT8 (Figure 6H). Classification averaging of all DMTs from C-SNAP tagged axonemes detected an additional density close to the inner junction, below the shorter part of the L-shaped structure that is missing from DMT5 to DMT8 in *mbo2* (Figures 7 and 8). The additional density marking the N-terminal SNAP tag is located above and slightly proximal of the C-terminal tag. It sits above the surface of the DMT, close to the tail domain of IDA *b*, but below the motor domain (Figures 7 and 8). The additional density marking the M-SNAP tag at amino acid 569 was detected beyond RS2, and near the surface of the DMT, between protofilaments A04 and A05 (Figures 7 and 8). This site is close to the distal end of the red structures detected as missing by cryo-ET of the *mbo2* mutant.

One possible interpretation of these observations is that the N-terminus of MBO2 is located close to IDA *b* and the polypeptide extends distally towards RS2; the MBO2 subunit then folds back proximally and turns downward towards the C-terminus, so that the N- and C-termini of one MBO2 subunit are both located near the same IDA *b* docking site. However, the MBO2 sequence is predicted to form several coiled-coil domains separated by regions whose structure cannot be predicted with high confidence (Figures 2A and 6A; Tam and Lefebvre, 2002). An alternative interpretation consistent with both the locations of the labels and the size and predicted structure of MBO2 polypeptide is that the MBO2 subunit adopts an extended confirmation spanning the 96-nm repeat, like that observed for the CCDC39/CCDC40 (FAP59/ FAP172) subunits (Oda *et al.*, 2014; Gui *et al.*, 2021). We favor this second interpretation based on the evidence described below.

### Model for the proposed location of the MBO2 subunit and its interaction with other complexes in the axoneme

**FIGURE 9:**
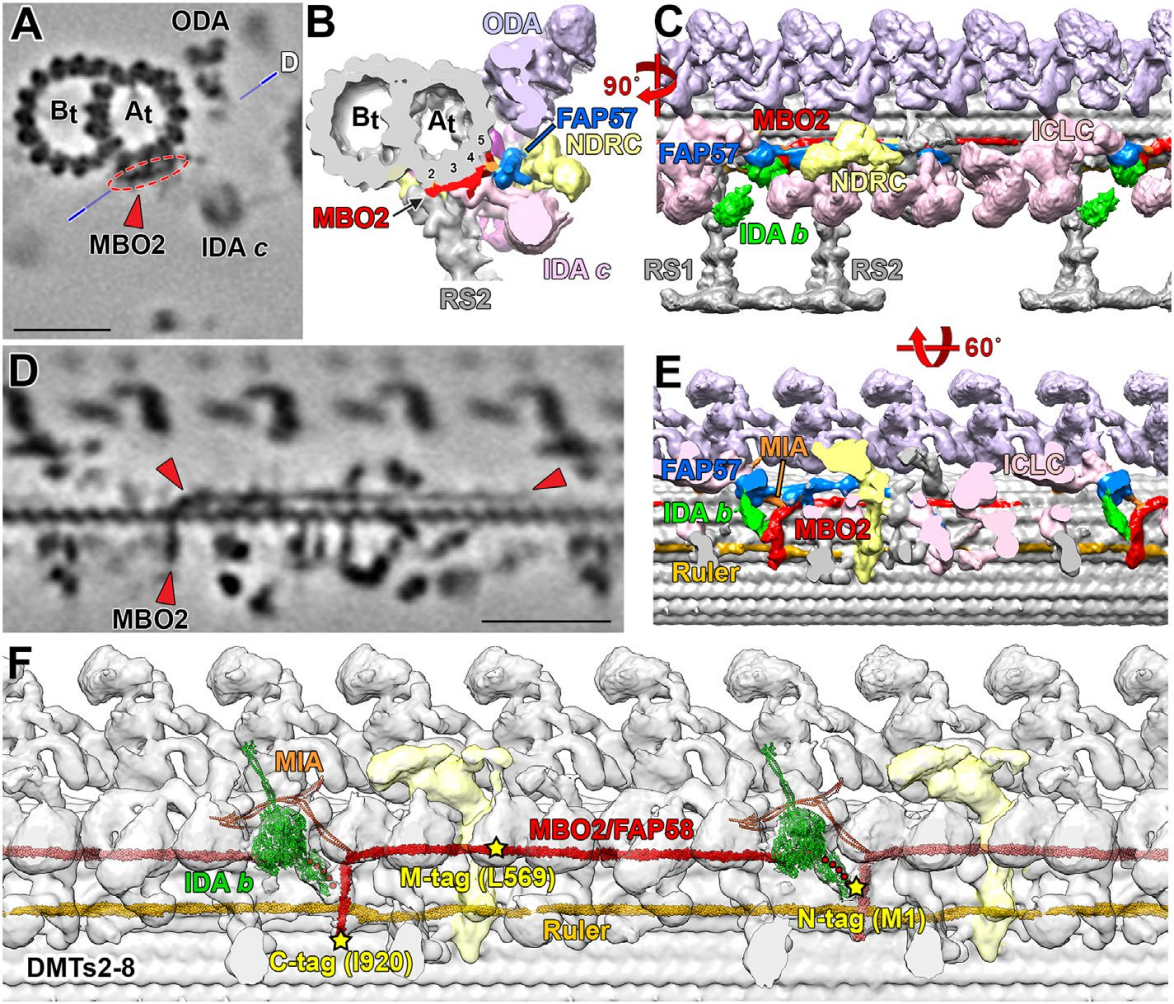
Summary images illustrating the proposed location of the MBO2/FAP58 heterodimer and its interactions with other regulatory complexes in the 96-nm repeat. (A and B) Tomographic slice and three-dimensional isosurface rendering of the DMT in cross-section in the region between RS1 and RS2 indicating a filamentous structure on the surface of DMT (circled in red in A and colored in red in B) running from the A02 protofilament to the cleft between protofilaments A04 and A05. (C) Rotating the isosurface model 90° along y-axis shows the position of the red structure below IDA *b* (in green) and the globular domain of FAP57 (in blue). The red structure continues along the surface of the DMT, running behind the N-DRC (yellow) and extending into the next 96-nm repeat. (D) A tomographic slice taken through the DMT along the plane illustrated by the line in (A) and (E) the corresponding isosurface model tilted 60 degrees along x-axis from the view in (C) shows the MBO2-associated, L-shaped structure running vertically from protofilament A02 to A04 and then bending and extending distally along the cleft between protofilaments A04 and A05 (indicated by red arrowheads in D and red structure in E). Clipping the isosurface rendering reveals the interactions of the L-shaped structure with the axoneme ruler (gold filamentous structure) located between protofilaments A02 and A03. The L-shaped structure extends upward near the base of IDA *b* (green) and the MIA complex (orange) and then turns distally, running below the FAP57 associated structure (blue) and N-DRC (yellow) and beyond RS2 and RS3S (unshaded) into the next 96-nm repeat, where it fades from view just below the I1 dynein. (F) A model for the proposed location of a MBO2/FAP58 heterodimer within the 96-nm repeat. AlphaFold2 was used to predict the structure of a MBO2/FAP58 heterodimer based on the Clustal W alignment of the MBO2, FAP58, and FAP189 polypeptide sequences (Supplemental Figure S6). The MBO2 structure was placed in the 96-nm repeat based on locations of the SNAP tags detected by streptavidin gold labeling (Figures 7 and 8). The density map of the DMT was obtained by classification averaging, and the class with intact IDA *b* structures is shown here. Because the N-terminal region of MBO2 is disordered, the filamentous structure shown in D and E could not be followed to the site predicted for N-terminal SNAP tag of MBO2. This flexible region (∼5.5 nm) is therefore indicated by a series of red dots. The proposed location for the MBO2/FAP58 heterodimer also agrees with the positioning of a FAP189 homodimer based on mapping residues 689–719 near the MIA complex and residues 384–480 near the FAP78 distal protrusion (Walton *et al.*, 2023). The AlphaFold2 models for the MIA complex and IDA *b* were added to illustrate how they are proposed to interact with the MBO2/FAP58 heterodimer (Yamamoto *et al.*, 2013; Walton *et al.*, 2023). Scale bars in (A) and (D) are 25 nm.

We identified a candidate L-shaped structure in our highest resolution images of WT *Chlamydomonas* axonemes that we propose as the likely location of the MBO2 polypeptide on DMTs 2-8 (Figure 9; Supplemental Table S5). We suggest that the C-terminal region of MBO2 corresponds to a filamentous structure that can be seen close to the surface of the A-tubule in cross-section, with the C-terminus located near protofilament A02 (Figure 9, A and B), consistent with the location of the C-terminal SNAP tag shown in Figure 7. The C-terminal region of the filament wraps around the A-tubule (circled in red/colored red in Figure 9, A and B) to a cleft between protofilaments A04 and A05. Rotating the images 90° along the y-axis (Figure 9, C and D) shows the position of the filament running vertically from protofilament A02 to A04 (red arrowheads in Figure 9D) and then turning distally and running along the surface of the DMT between protofilaments A04 and A05 to the red arrowhead on the right (Figure 9D). This filament is modeled in red in the isosurface rendering (Figure 9C). Tilting the isosurface rendering 60° as shown in Figure 9E, the MBO2-associated, L-shaped structure (red) interacts with the axoneme ruler (gold) at its C-terminus and then extends vertically, running below the base of IDA *b* (green) to contact the MIA complex (orange). The red structure turns distally to extend along the surface of the DMT, running below the FAP57 complex (blue) and N-DRC (yellow) and beyond the RS3S (unshaded) into the next 96-nm repeat, where it fades just below the I1 dynein, ∼5.5 nm from the predicted location of the N-terminal SNAP tag.

The L-shaped structures present in WT and missing in *mbo2* (Figures 4 and 7–9) are similar in location and organization to a L-shaped, coiled-coil structure described in cryo-EM studies of WT axonemes (see Figure 1 in Ma *et al.*, 2019; Extended Data Figure 4 in Gui *et al.*, 2021). This coiled-coil structure has recently been proposed as the location of FAP189 based on limited fitting of short regions of an AlphaFold2 predicted structure for a FAP189 homodimer (predicted in several sequence fragments) to a single particle, cryo-EM reconstruction of WT axonemes (Extended Data Figures 5 and 8 in Walton *et al.*, 2023). However, we have found that the decrease in MBO2 seen in all the *mbo* mutants is more closely correlated with a decrease in FAP58, whereas FAP189 levels are essentially unchanged or even increased in *mbo* mutant axonemes (Table 2; Supplemental Tables S4). This suggests that at least on DMTs 5–8, where the L-shaped structure is missing in *mbo2*, MBO2 and FAP58 form a heterodimer contained within the L-shaped, coiled-coil filament and that they also serve as a scaffold for the assembly of other polypeptides missing in *mbo* axonemes. We further hypothesize that the MBO2/FAP58 heterodimer and FAP189 homodimer perform distinct but complementary functions in stabilizing the binding of other proteins in the 96-nm repeat. MBO2/FAP58 and FAP189 may occupy similar sites on different DMTs or vary in proximal, medial, or distal regions. For instance, a FAP189 homodimer may form part of the L-shaped densities that remain on other DMTs in the *mbo2* mutant. The FAP58 and FAP189 subunits may also partially compensate for one another in different mutants such as *mbo1* where FAP189 appears to be elevated, similar to that proposed for different paralogues of the FAP57/FAP337 complex in *fap57* mutants (Lin *et al.*, 2019). Studies of a *fap189* mutant in *Chlamydomonas* will be required to resolve these questions.

To test the hypothesis that MBO2 and FAP58 form a heterodimer, we aligned the polypeptide sequences for MBO2, FAP58, and FAP189 using Clustal W (Supplemental Figure S4). We then used AlphaFold2 (Mirdita *et al.*, 2022) to predict the structure of a potential MBO2/FAP58 heterodimer using full length sequences. The resulting prediction shows extensive coiled-coil domains separated by short, unstructured regions, and slightly longer, unstructured regions at the N- and C-termini of both subunits (Supplemental Figure S5). We placed the proposed MBO2/FAP58 heterodimer within the 96-nm repeat based on the positions of the MBO2-SNAP tags detected by streptavidin gold labeling (Figures 7 and 8). Because the N-terminal region of MBO2 is thought to be disordered, we could not follow the filamentous structure completely to a site near the IDA *b* tail domain that is predicted to be the N-terminus of MBO2 (Figure 9), and so this region is represented by a series of red dots (Figure 9F). However, the positions of the M-SNAP tag at leucine 569 and the C-SNAP tag at isoleucine 920 are consistent with the lengths of the predicted coiled-coil domains (Figure 9F). The placement of the MBO2/FAP58 heterodimer is also consistent with the mapped positions of short segments of amino acids that are conserved between FAP189 and FAP58 (See Figure Legend 7 and Walton *et al.*, 2023) and recent chemical cross-linking experiments suggesting the presence of a CCDC146/CCDC147 heterodimer in *Tetrahymena* axonemes (McCafferty *et al.*, 2023).

FAP189 has been associated with subunits of the MIA complex and FAP57 complex based on chemical cross-linking and immunoprecipitation experiments (Yamamoto *et al.*, 2013). Given the sequence homology between FAP189 and FAP58 (∼80% sequence identity), it is likely the MBO2/FAP58 heterodimer also interacts with the MIA complex and the FAP57 complex (Figure 9E; Supplemental Figure S6), consistent with the proximity of these structures observed by cryo-ET. In *Chlamydomonas*, *fap57* mutants are associated with reductions in FAP57 and increases in two FAP57 paralogues (FBB7 and FAP331). The *fap57* mutants also display DMT-specific defects in assembly of IDA *g* and *d*. Increases in the levels of the FAP57 paralogues could partially compensate for the loss of FAP57 on certain DMTs in *fap57* mutants (Lin *et al.*, 2019). Interestingly, we have not seen any evidence that the *mbo* mutants disrupt the assembly of the MIA complex or FAP57 (Supplemental Table S4), even though we detected reductions in FAP331 and FAP337 in the *mbo* strains (Tables 1–3; Supplemental Table S4). Studies in *Tetrahymena* using chemical cross-linking, single particle cryo-EM and AlphaFold2 modeling have recently localized FAP337 to a “staple” formed by the coiled-coil heterodimer of CCDC96/CCDC113 (FAP184/FAP263) that is located distal to the N-DRC (Bazan *et al.*, 2021; Ghanaeian *et al.*, 2023). FAP337 also interacts with the base of IDA *d* and the coiled-coil domains of two FAP57 paralogues, CFAP57A/CFAP57C, and CFAP57A/C interacts in turn with bases of IDA *d* and *g* (Ghanaeian *et al.*, 2023). Collectively these observations illustrate how FAP57 and FAP337 (and their paralogues) could interconnect the MIA complex, the N-DRC, CCDC96/CCDC113, and stabilize the binding of IDA *d* and *g* (Lin *et al.*, 2019; Bazan *et al.*, 2021; Ghanaeian *et al.*, 2023).

In *Tetrahymena*, the CFAP57 paralogues also interact with an unidentified L-shaped, coiled-coil structure like the MBO2/FAP58/FAP189 structure described above (Ghanaeian *et al.*, 2023; Walton *et al.*, 2023). Our observations that FAP331 and FAP337 are reduced in *mbo* mutants are consistent with an interaction between these FAP57-associated proteins and the MBO2-associated L-shaped structure. We have not identified any defects on the assembly of IDA *d* (DHC2) and IDA *g* (DHC3, DHC7) in the *mbo* strains, but impact of any reductions the FAP57-associated proteins may be offset by the WT levels of FAP57 and numerous connections between the other structures in this region. We propose that the FAP57 paralogues are located in a similar location as FAP57 and that they serve a partially redundant and possibly DMT-specific function in *Chlamydomonas.* The proposed location of FAP57 and its paralogues relative to other structures in the 96-nm repeat is illustrated in Supplemental Figure S6.

### Interactions of the MBO2 complex with other structures in the axoneme and implications for motility

Previous work revealed that generation of ciliary and flagellar waveforms requires an asymmetric distribution of force generated by dynein arm activity along the cilia (Lin and Nicastro, 2018). MBO2 and its associated structures demonstrate that cilia have intrinsic asymmetry built into the structure of the axoneme, as well as their interconnections to multiple regulatory complexes, to facilitate both mechanical and chemical feedback control on dynein arm activity. A biophysical study of the beating patterns in *Chlamydomonas,* including the *mbo2* mutant, showed that the asymmetric ciliary beating waveform of *Chlamydomonas* WT could be mathematically separated into static and dynamic components (Geyer *et al*., 2016; Sartori *et al.*, 2016). That study concluded that *mbo2* lacks the static component and further predicted that there were defects in IDAs in the *mbo2* mutant (Geyer *et al*., 2016; Sartori *et al.*, 2016). Our findings strongly suggest that MBO2 and its associated IDA *b* structures contribute to the generation of the static component of the ciliary waveform in *Chlamydomonas*.

## MATERIALS AND METHODS

### Culture conditions, genetic analyses, and strain construction

Strains used in this study (Supplemental Table S1) were maintained on Tris-acetate phosphate (TAP) medium, but occasionally resuspended in liquid minimal medium or 10 mM HEPES, pH 7.6, to facilitate flagellar assembly and mating. Transformants were selected by cotransformation with pSI103 (encoding the *aphVIII* gene; Sizova *et al.*, 2001) or pHyg2 (encoding the *aphVII* gene; Berthold *et al.*, 2002) and plating on media containing 10 μg/ml paromomycin or hygromycin B.

### Epitope tagging of MBO2 and characterization of candidate dhc5 mutations

Purification of genomic and plasmid DNA, restriction enzyme digests, agarose gels, and PCR reactions, were performed as previously described (Lin *et al.*, 2019). All primers used for SNAP tagging of MBO2 and characterizing insertions into the *DHC5* gene are listed in Supplemental Table S2. The plasmid containing the wild-type *MBO2* gene tagged with a 2HA tag was previously described (Tam and Lefebvre, 2002), and the predicted polypeptide sequence is shown in Supplemental Figure S1. To make a construct encoding a SNAP tag at the N-terminus of MBO2, a 2033 bp gene fragment spanning two *Apa*I sites and encoding a codon optimized SNAP tag and first three exons and introns of *MBO2* was synthesized and cloned into pUC57 (Genewiz, Azenta Life Sciences, South Plainfield, NJ). This fragment was amplified by PCR with primers spanning the *Apa*I sites and complementary to *MBO2*. After gel purification, the SNAP-tagged fragment was assembled into a *Apa*I digested MBO2-HA plasmid using NEBuilder (New England Biolabs, Ipswich, MA). The final *N-SNAP-MBO2-HA* construct encodes an MBO2 polypeptide with a SNAP tag at its N-terminus and a 2-HA tag located between amino acids 885 and 886 of the original MBO2 sequence (Supplemental Figure S1).

To insert a SNAP tag near the middle of MBO2, a 1839 bp region between two *Kpn*I sites was amplified by PCR and subcloned into pGEM-T Easy (Promega, Madison, WI). This subclone was subjected to site-directed mutagenesis using the Q5 site-directed mutagenesis kit (New England Biolabs) to create a new *Hind*III site at the position encoding amino acid 569. A SNAP tag was amplified from a codon-optimized SNAP plasmid (Song *et al.*, 2015) using primers containing *Hind*III sites and complementary to MBO2, gel purified, and assembled into the *Hind*III site of the *Kpn*I subclone using NEBuilder. The tagged *Kpn*I subclone was then amplified with primers containing *Kpn*I sites, gel purified, and assembled into a *Kpn*I-digested MBO2-HA plasmid. The final *MBO2-M-SNAP-HA* construct encodes an MBO2 polypeptide with a SNAP tag located between amino acids 569 and 570 and a 2-HA tag between amino acids 885 and 886 of the original MBO2 sequence (Supplemental Figure S1).

To tag the C-terminus of MBO2, a 1018 gene fragment spanning two *Hpa*I sites and containing the last exon of MBO2 and codon optimized SNAP tag before the stop codon was synthesized and cloned into pUC57 (Genewiz). This fragment was then amplified using primers containing *Hpa*I sites, gel purified, and assembled into the *Hpa*I digested MBO2-HA plasmid. The final MBO2-C-SNAP construct encodes an MBO2 polypeptide with a SNAP tag at its C-terminus but lacking the HA tag (Supplemental Figure S1).

All new constructs were verified by sequencing (GeneWiz), analyzed using the Sequencher (Gene Codes, Ann Arbor, MI) and MacVector (Apex, NC) software packages, and linearized with *Eco*R1 or *Bam*H1 before cotransformation into *mbo2-4*.

### Phase contrast microscopy and measurements of swimming velocity

Motility phenotypes were assessed by phase contrast microscopy using a 20x or 40x objective on a Zeiss Axioskop microscope. Measurements of swimming velocities were made from recordings using a Rolera-MGi EM-CCD camera (Q-imaging, Surrey, BC, Canada) and the Metamorph software (Molecular Devices, San Jose, CA; VanderWaal *et al.*, 2011; Bower *et al.*, 2013, 2018; Reck *et al.*, 2016). At least three independent experiments were performed for each strain. Data are presented as the mean ± SD using the student’s *t* test. Images of forward movement were obtained by collecting 1 s exposures of cells imaged with the 20x objective. Selected images were cropped, rotated, and labeled in Image J and Adobe Photoshop (San Jose, CA).

### Fractionation of axonemes, SDS–PAGE, Western blot, and tandem mass spectrometry (MS/MS) analysis of gel bands

*Chlamydomonas* whole cell lysates, flagella, and axonemes were prepared as previously described (Witman, 1986; Bower *et al.*, 2013, 2018; Reck *et al.*, 2016) using 0.1–1.0% Nonidet-P-40 to remove membrane plus matrix proteins. Purified axonemes were resuspended in HMEEN (10 mM HEPES, pH 7.4, 5 mM MgSO4, 1 mM EGTA, 0.1 mM EDTA, 30 mM NaCl) plus 1 mM dithiothreitol (DTT) and 0.1 μg/ml protease inhibitors (leupeptin, aprotinin, pepstatin), and extracted with HMEEN containing 0.6 M NaCl, 0.2 M NaI, 0.4 M NaI, or 0.6 M NaI. Some samples were also extracted with 0.1–0.7% Sarkosyl. The 0.6 M extracts from *pf28* (containing IDAs) were diluted 10-fold and fractionated by Mono-Q ion-exchange FPLC chromatography (Gardner *et al.*, 1994). Samples were separated on 5–15% polyacrylamide gradient gels and silver stained or transferred to Immobilon P and probed with different antibodies (Supplemental Figure S2; Supplemental Table S3; Bower *et al.*, 2013, 2018). A subset of FPLC fractions were analyzed by SDS–PAGE and MS/MS to identify polypeptides that cofractionate with DHC5. Selected bands were excised and analyzed as previously described (Lin *et al.*, 2019).

Because DHCs vary widely in abundance, purified axonemes were also fractionated by SDS–PAGE, stained briefly with Coomassie blue, and the DHC region was excised from the gel to improve the signal to noise. Following extraction and trypsin digestion, three to five replicates per sample were analyzed by MS/MS, and both the total number of peptides and total number of assigned spectra per HC isoform were determined. The relative abundance of each DHC was estimated by spectral counting (Zhu *et al.*, 2010) and expressed as a percentage of the total spectra identified for the 1-alpha and 1-beta DHCs of the I1 dynein as previously described (Bower *et al.*, 2013, 2018). The DHCs were also identified and quantified using the SEQUEST algorithm (Eng *et al.*, 1994) and Proteome Discover 2.3 (Thermo Fisher Scientific). For internal calibration of the peptide masses, the recalibration node was used with a 20 ppm mass tolerance and carbamidomethyl cysteine as a fixed modification. For protein identification, we used the *Chlamydomonas reinhardtii* v5.6 protein FASTA database concatenated with a common lab contaminant database (www.thegpm.org/crap/) and the following SEQUEST search parameters: semitrypsin, two missed cleavage sites, minimum peptide length six, precursor mass tolerance 12 ppm, fragment mass tolerance 0.1 Da, dynamic modifications: oxidation of methionine, deamidation of asparagine and glutamine and pyro-glutamic acid modification of N-terminal glutamine, and carbamidomethyl cysteine as a fixed modification. We used the Percolator algorithm with a concatenated target-decoy database approach to control the false discovery rate (FDR; Brosch *et al.*, 2009; Spivak *et al.*, 2009). Each sample was analyzed in triplicate and quantified using the label free quantification workflow that includes steps for feature extraction, chromatographic alignment, peptide mapping to features, protein abundance calculation, normalization, protein relative abundance ratio calculation and hypothesis testing for significance of relative fold change. For each file, we applied the untargeted Minora Feature Detector algorithm, which is similar to the match between runs setting in MaxLFQ (Cox *et al.*, 2014). Peptides were mapped to retention time-aligned consensus features across samples with the requirement that at least one sample contains a peptide spectral match. For each sample, protein abundances were calculated by summing abundances of consensus features for related peptides. We normalized protein abundances for each sample using two proteins: DHC1 (178 distinct peptide sequences) and DHC10 (152 distinct peptide sequences). We set the hypothesis test method to ANOVA and report *p* values that were adjusted using the Benjamini-Hochberg correction for FDR.

### Preparation of samples for iTRAQ or TMT labeling and MS/MS analysis of whole axonemes

iTRAQ labeling: Axonemes were washed in 10 mM HEPES pH 7.4 to remove salt, DTT, and protease inhibitors, then resuspended in 0.5 M triethylammonium bicarbonate pH 8.5 and processed for trypsin digestion and iTRAQ labeling as described in detail (Bower *et al.*, 2013, 2018; Reck *et al.*, 2016). Duplicate aliquots of axonemes (50–60 μg each) from each strain were reacted with four-plex iTRAQ reagents (114–117, AB Sciex, Foster City, CA) to obtain two technical replicates per biological sample. The four labeled aliquots were mixed and processed to remove excess trypsin, unreacted iTRAQ reagents, and buffer. The combined sample (containing two control aliquots with different iTRAQ labels and two mutant aliquots with different iTRAQ labels) was fractionated offline using high pH, C18 reversed phase chromatography (Reck *et al.*, 2016). Approximately 500 ng of each peptide fraction was analyzed by LC–MS on a Velos Orbitrap mass spectrometer (Thermo Fisher Scientific, Waltham MA). Online capillary LC, MS/MS, database searching, and protein identification were performed as previously described (Lin-Moshier *et al.*, 2013; Reck *et al.*, 2016) using ProteinPilot software version 4.5 or 5.0 (AB Sciex, Foster City, CA) and version 5.5 of the *Chlamydomonas* genome database (https://phytozome.jgi.doe.gov/pz/portal.html). The bias factors for all samples were normalized to alpha and beta tubulin (Reck *et al.* 2016). The relative amount of protein in each aliquot was compared with that present in the control aliquot to obtain a protein ratio. The WT/WT or HA/HA ratios indicated the variability in labeling and protein loading between technical replicates of the same sample (typically less than 10% for all proteins). Two iTRAQ experiments with independent biological replicates were performed for *mbo1*, *mbo2*, and *pf12.* Between 689 and 919 proteins were identified at a 1% FDR in each experiment. The protein lists were filtered using a minimum of six peptides per protein, and those proteins that were significantly reduced (*P* < 0.05) in all samples using Benjamini–Hochberg correction were further analyzed. We also reanalyzed proteomics data from other iTRAQ experiments using two biological replicates of *ida8* axonemes with defects in the FAP57 complex (Lin *et al.*, 2019), and two biological replicates of *pf9-2; pf28* axonemes, a double mutant that lacks ODAs and I1 dynein and assembles short (∼2.9 μm) flagella (Porter *et al.*, 1992; Hwang *et al*., 2024).

TMT labeling: The proteins altered in *mbo2* axonemes were analyzed in a third experiment using three independent biological replicates of WT, *mbo2*, and *MBO2-HA* axonemes and TMT16 plex labeling. We also analyzed axonemes from WT and a *pf12; fap 20* mutant in another TMT experiment. Samples were digested and labeled with TMT reagents, dried down, and then fractionated offline as described above. Approximately ∼300–600 nanograms of each fraction was analyzed by capillary LC–MS with a Dionex UltiMate 3000 RSLC nano system on-line with an Orbitrap Eclipse mass spectrometer (Thermo Fisher Scientific) with FAIMS (high-field asymmetric waveform ion mobility) separation as described by Weise *et al.*, 2023 with minor modifications. The LC profile was 5 to 8% solvent B at 2.5 min, 21% B at 135 min, 34% B at 180 min and 90% B at 182 min with a flowrate of 315 nl/min, where solvent A was 0.1% formic acid in water and solvent B was 0.1% formic acid in ACN. The MS2 settings were: 0.7 Da quadrupole isolation window, 38% fixed collision energy, Orbitrap detection with 50K resolution at 200 *m/z*, first mass fixed at 110 *m/z*, 150 msec max injection time, 250% (1.25 × 10E5) AGC, 45 s dynamic exclusion duration with ± 10 ppm mass tolerance and exclusion lists were not shared among compensation voltages.

The peptide output from the tandem MS was processed using SEQUEST algorithm in Proteome Discoverer 2.5. The *Chlamydomonas reinhardtii* protein sequence database was downloaded from https://phytozome-next.jgi.doe.gov/info/Creinhardtii_v5.6 and merged with two additional sequences, DRC1 and FAP58, plus a common lab contaminant protein database (www.thegpm.org/cRAP/index.html) (16,631 total protein sequences). The database search parameters were as previously described (Weise *et al.*, 2023) with the exception that the fragment ion tolerance was 0.08 Da. Peptides and proteins were identified using a 1% FDR and the Percolator algorithm (Käll *et al.*, 2008) in Proteome Discover and quantified as described in Weise *et al.*, 2023, with the exception that normalization was performed using alpha and beta tubulin. The data were also reanalyzed with the latest version of the *Chlamydomonas* sequence database (JGI v6.1, 16883 protein coding genes). FAP58 was annotated in early versions of the genome as Cre13.g584300, dropped for unknown reasons in v5.6, and renumbered as Cre13.g801543 in v6.1. We have included both JGI genome ID numbers here. The software used for analysis of TMT labeled samples differs in several ways from the Protein Pilot software used for analysis of iTRAQ labeled samples, but both methods provide estimates of relative protein ratios based on the quantification of isobaric tags.

### Cryo-sample preparation, cryo-electron tomography, and image processing

The purified axoneme pellet was resuspended in HMEEK buffer (30 mM HEPES, pH7.4, 5 mM MgSO_4_, 1 mM EGTA, 0.1 mM EDTA, 25 mM KCl), and the suspension was directly used for cryo-sample preparation. For visualization of the SNAP tags, streptavidin nanogold labeling was performed on axonemes from strains rescued with SNAP-tagged versions of the MBO2 as previously described (Song *et al.*, 2015). Briefly, 1 µl of 1 mM BG-(PEG)12-biotin (New England Biolabs; PEG linker available on request) was added to 200 µl of axonemes. A control sample was also prepared without added BG-(PEG)12-biotin. Both suspensions were incubated overnight at 4°C, followed by three cycles of resuspension with HMEEK buffer and centrifugation at 10,000 g for 10 min at 4°C. The axoneme pellets were resuspended in 200 µl of buffer, and then either 5 µl of 1.4-nm-sized streptavidin nanogold particles (strep-Au, Nanoprobes) or 5 µl of buffer were added, and the two suspensions were incubated at 4°C in the dark for 3 h with rotation. The samples were then diluted with 1 ml of HMEEK buffer, pelleted by centrifugation at 10,000 g for 10 min at 4°C, carefully resuspended in 200 µl of HMEEK buffer, and used for cryo-sample preparation.

Cryo-sample preparation, cryo-ET and image processing were done as previously described (Nicastro *et al.*, 2006; Nicastro, 2009; Heuser *et al.*, 2009; Lin *et al.*, 2014). Briefly, Quantifoil copper grids (Quantifoil Micro Tools, Jena, Germany) with a holey carbon film (R2/2, 200 mesh) were glow discharged for 30 s at –40 mA and loaded with 3 µl of axoneme sample and 1 µl of BSA-coated, fivefold concentrated 10-nm colloidal gold (Sigma-Aldrich, St. Louis, MO; Iancu *et al.*, 2006). After brief mixing, grids were blotted from the back side with filter paper (Whatman #1) for 1.5–3 s and plunge frozen in liquid ethane using a home-made plunger. Vitrified samples were either loaded onto a cryo holder (Gatan, *Pleasanton*, CA) and transferred to a Tecnai F30 or assembled into autogrids and transferred to a Titan Krios transmission electron microscope (Thermo Fisher Scientific, Waltham, MA) for imaging. Single-axis tilt series of noncompressed, intact axonemes were acquired using the software package SerialEM (Mastronarde, 2005). Typically, 50 to 70 images were recorded for each tilt series while the specimen was tilted from about –65 to +65° in 1.5 to 2.5° increments. A dose-symmetric scheme was applied for data collection (Hagen *et al.*, 2017). The magnification was set to 13,500X (∼1 nm effective pixel size) with –6 to –8 µm defocus (for Tecnai F30) or 26,000X (∼0.55 nm effective pixel size) with –0.5 µm defocus using a Volta phase plate (Danev *et al.*, 2014) (for Titan Krios). The microscope was operated in low dose mode at 300 keV and the accumulative electron dose of the sample was restricted to ∼100 e/Å^2^ to minimize radiation damage. Electron micrographs were recorded with a 2k x 2k CCD camera (on Tecnai F30) or with a 4k x 4k K2 electron direct camera. The K2 camera was operated in counting mode (0.4-s exposure time per frame and 15 frames per tilt series with a dose rate of eight e^–^/p/s. Both TEMs were equipped with a postcolumn energy filter (Gatan, *Pleasanton*, CA) that was operated in zero-loss mode with a slit width of 20 eV.

The raw frames from the K2 camera were aligned for motion-correction with a script from the IMOD software (Kremer *et al.*, 1996). Three-dimensional tomograms were reconstructed using fiducial alignment of the tilt series images and weighted backprojection using IMOD. Subtomograms containing the 96-nm repeat units were further aligned and averaged using PEET (Nicastro *et al.*, 2006), resulting in averaged three-dimensional structures with compensated missing wedge effect, reduced noise, and thus increased resolution. For doublet-specific averaging, the nine outer DMTs were identified based on DMT-specific features (Bui *et al.*, 2012; Lin *et al.*, 2012), and repeats from individual DMTs were averaged. To further analyze structural defects that appeared heterogeneous or to identify the sites labeled with nanogold particles, classification analyses were performed on the aligned sub-tomograms using the PEET program (Heumann *et al.*, 2011). Appropriate masks were applied to focus the classification analysis on specific regions of interest, and sub-tomograms containing the same structures were grouped into class averages. The structures were mapped onto their respective locations in the raw tomograms to determine the distribution of the different classes. The numbers of tomograms and sub-tomograms that were analyzed and the resolutions of the resulting averages are summarized in Supplemental Table S5. The resolution was estimated at the center of the sub-tomogram volume using the Fourier shell correlation method with a criterion of 0.5. The structures were visualized as two-dimensional tomographic slices and three-dimensional isosurface renderings using IMOD and UCSF Chimera (Pettersen *et al.*, 2004), respectively. The structure of the MBO2/FAP58 heterodimer was predicted using the AlphaFold 2 software (Jumper *et al.*, 2021; Mirdita *et al.*, 2022). To place the pseudoatomic model into the L-shaped structure, the two longest coiled-coil regions were arranged into one long filament and the shorter coiled-coil tilted into an about 90° angle relative to the filament (Figure 7F).

**Figure:** Movie S1 **Visualization of the averaged doublet microtubule from a *Chlamydomonas* WT axoneme**. The video was generated using the tomogram obtained from the highest resolution WT axoneme sample (pWT, see Supplemental Table S5). The tomographic slices in cross‐sectional orientation (from proximal to distal direction) and longitudinal orientation (moving from the B‐tubule through the A‐tubule) are displayed at the beginning of the video. The positions and densities corresponding to the ruler and MBO2 structures are indicated by labeling and showing their 3D isosurface renderings. The isosurface rendering of the entire averaged structure is shown in different orientations, with emphasis on the L‐shaped MBO2 associated structure. The localizations of the Au‐SNAP tags at the N‐, M‐ and Cterminal regions are highlighted by yellow stars, and the flexible N‐terminal region of MBO2 that is not visible in the subtomogram averages is indicated by a red dotted line.

## Supplementary Material





## Abbreviations used:

CSC: Calmodulin- and spoke-associated complex
CCDC: Coiled coil domain containing
CP: Central pair
CliP: *Chlamydomonas* Library Project
DIC: Differential interference contrast
DHC: Dynein heavy chain
ET: Electron tomography
FAP: Flagellar associated polypeptide
FPLC: Fast protein liquid chromatography
HC: Heavy chain
HA: Hemagglutinin
IDA: Inner dynein arm
IC: Intermediate chain
IFT: Intraflagellar transport
iTRAQ: Isobaric tag for relative and absolute quantitation
LC: Light chain
MBO: Move backwards only
N-DRC: Nexin-dynein regulatory complex
ODA: Outer dynein arm
*pf*: Paralyzed flagella
PEET: Particle Estimation for Electron Tomography
PCR: Polymerase chain reaction
PCD: Primary ciliary dyskinesia
RS: Radial spoke
RSP: Radial spoke protein
RS3S: Radial spoke 3 stand-in
MS/MS: Tandem mass spectrometry
TMT: Tandem mass tag
TEM: Transmission electron microscopy
TAP: Tris-acetate-phosphate
WT: Wild-type
