# Supplementary material for: The MBO2/FAP58 heterodimer stabilizes assembly of inner arm dynein b and reveals axoneme asymmetries involved in ciliary waveform
Source: Mol Biol Cell. 2024 Apr 19;35(5):ar72. doi: 10.1091/mbc.E23-11-0439 (PMC11151096; doi:10.1091/mbc.E23-11-0439)
Supplement: Supplementary file 2 [file mbc-35-ar72-s001.pdf]

Supplemental Materials

Molecular Biology of the Cell

Fu *et al*.

Supplemental Information for

The MBO2/FAP58 heterodimer stabilizes assembly of inner arm dynein *b* and reveals axoneme asymmetries involved in ciliary waveform

Gang Fu[1], Katherine Augspurger[2], Jason Sakizadeh[2], Jaimee Reck[2], Raqual Bower[2], Douglas Tritschler[2], Long Gui[1], Daniela Nicastro[1*], and Mary E. Porter[2*]

[1] Department of Cell Biology, University of Texas Southwestern Medical Center, 6000 Harry Hines Blvd, Dallas, TX 75390

[2] Department of Genetics, Cell Biology, and Genetics, University of Minnesota, Minneapolis, MN 55455

*Corresponding authors

Mary E. Porter, Email: porte001@umn.edu

Daniela Nicastro, Email: daniela.nicastro@utsouthwestern.edu

This PDF file includes:

Figures S1 to S6

Tables S1 to S3, S5

References for supplemental information citations

Other supplementary materials for this manuscript include:

Table S4. MBO2 associated MS/MS data.xslx

Video S1. Visualization of the averaged doublet microtubule.

Figure S1. Predicted amino acid sequences of MBO2 and its tagged variants. The MBO2 sequence is shown in black, and the peptide sequence used for antibody production (Tam and Lefebvre, 2002) is underlined. The 2-HA-tag is shown in blue, and the SNAP-tags are shown in red.

MBO2 (104.3 kD)
MDDDILGLEDVTASEAFKIIYELSKASKIDADRSDAAKAQFTLLHSTLLQALQAERDLLDQAKALKRQKEEQEAAITGAGGVPGADDIDQLREDVEA
ALGEAALAQERQQLLQLEVTDLQRQRNDLGARMEELAAEHAAALQPVIAQARGEAAALVDELDEERRRVEAAHGELEDMKSKLAAVQEEIEAMG
ETKAVERANLVKVDPLPDKARRQSEAVAAILKSVQGQLESMNARYGEHDTQYKTAAEREQQLTEEHTKMLTALERGRVQVESKARHADDVRKD
VELASIEADKILADQVELDLRVKNLALELKAESDHLNRRQREKELMLRQYRVADQQLKDARDMLPNLKFQVEQMHRDVNTLEARRRAQSRELSD
VKRELDIQMAAFLHEEADGKEKVALFQLTYKEVAALEAELAALKREEAERDTILRDLGSQRDRVALAIAQKLSKVKDVQMTSRIKEVELAELKKIRK
EVGRRIRDFEKLYDLVKNQRNKFVNLIQAASQSTTEMKDKLKVLQNELDILQNEVGIKDKLLQQQHTQHAANIAERDQLRVELGRLGMVFRDKQA
VVDEQIAEVDKLNAIINGCEKEMLRLKKQYELVIEARNYTGIMLIDRNDELCVLYEKANILDEVIKSGQLELMRREDEARLLRLEVGELERSIGVTRRL
VPSVPLLDNDVAALQKALFEARREAEALSLALENPSNQGRWRLLEGKIPDREELSAKIQALEERLNDKKEQLLEKELILEEITSLSDKLRVQAAEGR
ADTLELAQRVNEYQSKLRAVTRKIMATVSELSMYQASALKLGAEKEELEGAVSLASQRLEAGEPPTDDAEREWYRLERERHTVDAMAEERRAVA
AALDARVAEVQSTAEPRPNAYIPEQLGIPKPYGSFAPFKPQEAGSTMRHIRKPSPKEVVI

MBO2-2HA (106.6 kD)
MDDDILGLEDVTASEAFKIIYELSKASKIDADRSDAAKAQFTLLHSTLLQALQAERDLLDQAKALKRQKEEQEAAITGAGGVPGADDIDQLREDVEA
ALGEAALAQERQQLLQLEVTDLQRQRNDLGARMEELAAEHAAALQPVIAQARGEAAALVDELDEERRRVEAAHGELEDMKSKLAAVQEEIEAMG
ETKAVERANLVKVDPLPDKARRQSEAVAAILKSVQGQLESMNARYGEHDTQYKTAAEREQQLTEEHTKMLTALERGRVQVESKARHADDVRKD
VELASIEADKILADQVELDLRVKNLALELKAESDHLNRRQREKELMLRQYRVADQQLKDARDMLPNLKFQVEQMHRDVNTLEARRRAQSRELSD
VKRELDIQMAAFLHEEADGKEKVALFQLTYKEVAALEAELAALKREEAERDTILRDLGSQRDRVALAIAQKLSKVKDVQMTSRIKEVELAELKKIRK
EVGRRIRDFEKLYDLVKNQRNKFVNLIQAASQSTTEMKDKLKVLQNELDILQNEVGIKDKLLQQQHTQHAANIAERDQLRVELGRLGMVFRDKQA
VVDEQIAEVDKLNAIINGCEKEMLRLKKQYELVIEARNYTGIMLIDRNDELCVLYEKANILDEVIKSGQLELMRREDEARLLRLEVGELERSIGVTRRL
VPSVPLLDNDVAALQKALFEARREAEALSLALENPSNQGRWRLLEGKIPDREELSAKIQALEERLNDKKEQLLEKELILEEITSLSDKLRVQAAEGR
ADTLELAQRVNEYQSKLRAVTRKIMATVSELSMYQASALKLGAEKEELEGAVSLASQRLEAGEPPTDDAEREWYRLERERHTVDAMAEERRAVA
AALDARVAEVQSTAEPRPNAYIPEQRYPYDVPDYAYPYDVPDYADLGIPKPYGSFAPFKPQEAGSTMRHIRKPSPKEVVI

N-SNAP-MBO2-2HA (126.6 kD)
MDKDCEMKRTTLDSPLGKLELSGCEQGLHEIKLLGKGTSAADAVEVPAPAAVLGGPEPLMQATAWLNAYFHQPEAIEEFPVPALHHPVFQQESF
TRQVLWKLLKVVKFGEVISYQQLAALAGNPAATAAVKTALSGNPVPILIPCHRVVSSSGAVGGYEGGLAVKEWLLAHEGHRLGKPGLGPAGIGAP
GSMDDDILGLEDVTASEAFKIIYELSKASKIDADRSDAAKAQFTLLHSTLLQALQAERDLLDQAKALKRQKEEQEAAITGAGGVPGADDIDQLREDV
EAALGEAALAQERQQLLQLEVTDLQRQRNDLGARMEELAAEHAAALQPVIAQARGEAAALVDELDEERRRVEAAHGELEDMKSKLAAVQEEIEA
MGETKAVERANLVKVDPLPDKARRQSEAVAAILKSVQGQLESMNARYGEHDTQYKTAAEREQQLTEEHTKMLTALERGRVQVESKARHADDVR
KDVELASIEADKILADQVELDLRVKNLALELKAESDHLNRRQREKELMLRQYRVADQQLKDARDMLPNLKFQVEQMHRDVNTLEARRRAQSREL
SDVKRELDIQMAAFLHEEADGKEKVALFQLTYKEVAALEAELAALKREEAERDTILRDLGSQRDRVALAIAQKLSKVKDVQMTSRIKEVELAELKKI
RKEVGRRIRDFEKLYDLVKNQRNKFVNLIQAASQSTTEMKDKLKVLQNELDILQNEVGIKDKLLQQQHTQHAANIAERDQLRVELGRLGMVFRDK
QAVVDEQIAEVDKLNAIINGCEKEMLRLKKQYELVIEARNYTGIMLIDRNDELCVLYEKANILDEVIKSGQLELMRREDEARLLRLEVGELERSIGVT
RRLVPSVPLLDNDVAALQKALFEARREAEALSLALENPSNQGRWRLLEGKIPDREELSAKIQALEERLNDKKEQLLEKELILEEITSLSDKLRVQAA
EGRADTLELAQRVNEYQSKLRAVTRKIMATVSELSMYQASALKLGAEKEELEGAVSLASQRLEAGEPPTDDAEREWYRLERERHTVDAMAEERR
AVAAALDARVAEVQSTAEPRPNAYIPEQRYPYDVPDYAYPYDVPDYADLGIPKPYGSFAPFKPQEAGSTMRHIRKPSPKEVVI

MBO2-M-SNAP-2HA (126.7 kD)
MDDDILGLEDVTASEAFKIIYELSKASKIDADRSDAAKAQFTLLHSTLLQALQAERDLLDQAKALKRQKEEQEAAITGAGGVPGADDIDQLREDVEA
ALGEAALAQERQQLLQLEVTDLQRQRNDLGARMEELAAEHAAALQPVIAQARGEAAALVDELDEERRRVEAAHGELEDMKSKLAAVQEEIEAMG
ETKAVERANLVKVDPLPDKARRQSEAVAAILKSVQGQLESMNARYGEHDTQYKTAAEREQQLTEEHTKMLTALERGRVQVESKARHADDVRKD
VELASIEADKILADQVELDLRVKNLALELKAESDHLNRRQREKELMLRQYRVADQQLKDARDMLPNLKFQVEQMHRDVNTLEARRRAQSRELSD
VKRELDIQMAAFLHEEADGKEKVALFQLTYKEVAALEAELAALKREEAERDTILRDLGSQRDRVALAIAQKLSKVKDVQMTSRIKEVELAELKKIRK
EVGRRIRDFEKLYDLVKNQRNKFVNLIQAASQSTTEMKDKLKVLQNELDILQNEVGIKDKLLQQQHTQHAANIAERDQLRVELGRLGMVFRDKLM
DKDCEMKRTTLDSPLGKLELSGCEQGLHEIKLLGKGTSAADAVEVPAPAAVLGGPEPLMQATAWLNAYFHQPEAIEEFPVPALHHPVFQQESFTR
QVLWKLLKVVKFGEVISYQQLAALAGNPAATAAVKTALSGNPVPILIPCHRVVSSSGAVGGYEGGLAVKEWLLAHEGHRLGKPGLGPAGIGAPGS
GAVVDEQIAEVDKLNAIINGCEKEMLRLKKQYELVIEARNYTGIMLIDRNDELCVLYEKANILDEVIKSGQLELMRREDEARLLRLEVGELERSIGVT
RRLVPSVPLLDNDVAALQKALFEARREAEALSLALENPSNQGRWRLLEGKIPDREELSAKIQALEERLNDKKEQLLEKELILEEITSLSDKLRVQAA
EGRADTLELAQRVNEYQSKLRAVTRKIMATVSELSMYQASALKLGAEKEELEGAVSLASQRLEAGEPPTDDAEREWYRLERERHTVDAMAEERR
AVAAALDARVAEVQSTAEPRPNAYIPEQRYPYDVPDYAYPYDVPDYADLGIPKPYGSFAPFKPQEAGSTMRHIRKPSPKEVVI

MBO2-C-SNAP (124.2 kD)
MDDDILGLEDVTASEAFKIIYELSKASKIDADRSDAAKAQFTLLHSTLLQALQAERDLLDQAKALKRQKEEQEAAITGAGGVPGADDIDQLREDVEA
ALGEAALAQERQQLLQLEVTDLQRQRNDLGARMEELAAEHAAALQPVIAQARGEAAALVDELDEERRRVEAAHGELEDMKSKLAAVQEEIEAMG
ETKAVERANLVKVDPLPDKARRQSEAVAAILKSVQGQLESMNARYGEHDTQYKTAAEREQQLTEEHTKMLTALERGRVQVESKARHADDVRKD
VELASIEADKILADQVELDLRVKNLALELKAESDHLNRRQREKELMLRQYRVADQQLKDARDMLPNLKFQVEQMHRDVNTLEARRRAQSRELSD
VKRELDIQMAAFLHEEADGKEKVALFQLTYKEVAALEAELAALKREEAERDTILRDLGSQRDRVALAIAQKLSKVKDVQMTSRIKEVELAELKKIRK
EVGRRIRDFEKLYDLVKNQRNKFVNLIQAASQSTTEMKDKLKVLQNELDILQNEVGIKDKLLQQQHTQHAANIAERDQLRVELGRLGMVFRDKQA
VVDEQIAEVDKLNAIINGCEKEMLRLKKQYELVIEARNYTGIMLIDRNDELCVLYEKANILDEVIKSGQLELMRREDEARLLRLEVGELERSIGVTRRL
VPSVPLLDNDVAALQKALFEARREAEALSLALENPSNQGRWRLLEGKIPDREELSAKIQALEERLNDKKEQLLEKELILEEITSLSDKLRVQAAEGR
ADTLELAQRVNEYQSKLRAVTRKIMATVSELSMYQASALKLGAEKEELEGAVSLASQRLEAGEPPTDDAEREWYRLERERHTVDAMAEERRAVA
AALDARVAEVQSTAEPRPNAYIPEpGIPKPYGSFAPFKPQEAGSTMRHIRKPSPKEVVIMDKDCEMKRTTLDSPLGKLELSGCEQGLHEIKLLGKG
TSAADAVEVPAPAAVLGGPEPLMQATAWLNAYFHQPEAIEEFPVPALHHPVFQQESFTRQVLWKLLKVVKFGEVISYQQLAALAGNPAATAAVKT
ALSGNPVPILIPCHRVVSSSGAVGGYEGGLAVKEWLLAHEGHRLGKPGLGPAGIGAPGS

Figure S2. Protein samples used for MS/MS analysis and quantification of DHC content.

(A) Western blot of three independent replicates of WT, *mbo2*, and *MBO2-HA* axonemes that were labeled using TMT isobaric tags and subjected to MS/MS. Blot was probed with antibodies to MBO2 and DIC2 (IC69). **(B)** A 0.6M NaCl extract of *pf28* axonemes was fractionated by FPLC. The fraction containing the peak of IDA *b*/DHC5 was analyzed by SDS-PAGE and silver stained, and indicated bands were excised and analyzed by MS/MS. **(C)** Axonemes from WT, *mbo2*, and three SNAP-tagged rescued strains were analyzed by SDS-PAGE and stained with Coomassie Blue. The region containing the DHCs (outlined in black) was excised and analyzed by MS/MS. **(D)** The relative abundance of DHCs from the samples shown in (C) was determined by spectral counting. Only DHC5 was significantly reduced in *mbo2* (red arrowhead). DHC content was also quantified using the tools available in Proteome Discover (Supplemental Table S4).

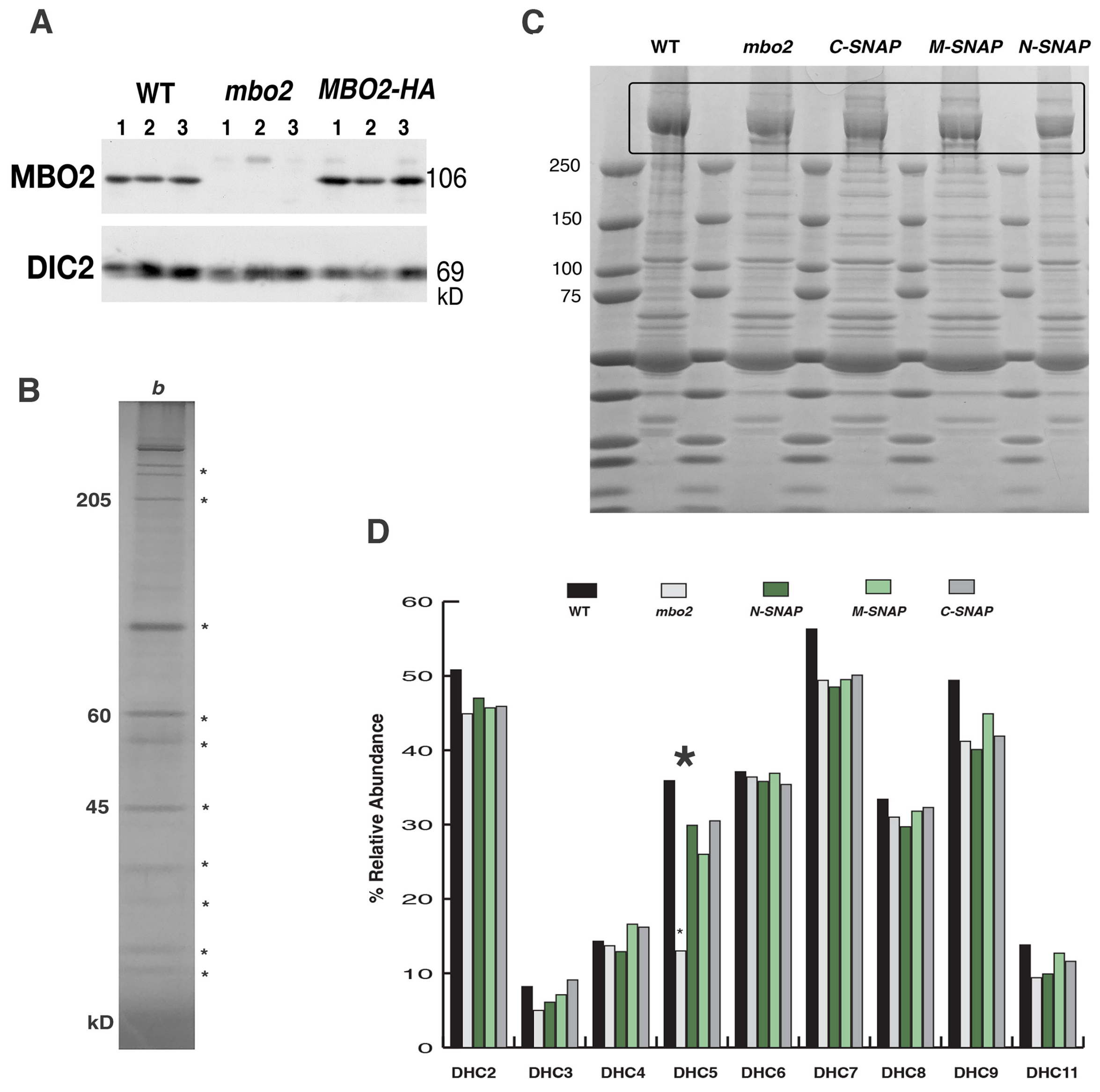

Figure S3. Loss of the DHC5 motor domain does not significantly alter forward swimming velocities.

(A) Four *dhc5* strains generated by insertional mutagenesis were tested by PCR to confirm insertion sites in the 5'end of the *DHC5* gene encoding the N-terminal tail domain of DHC5. A small region of Chromosome 13 (Cre13) is shown here indicating the predicted plasmid insertion sites and the location of the PCR primers used to confirm the sites. **(B)** Genomic DNA was isolated from a control strain (*cw15*) and four *dhc5* candidates (referred to as *248, 264, 438, 953* based on the last 3 digits of the strain number) and subjected to PCR with control primers and experimental primers shown in (**A)** and Supplemental Table S2. **(C)** Schematic diagram of the DHC5 polypeptide showing the approximate location of the six AAA ATPase domains (gray), the microtubule binding domain (MTB, black), and the plasmid insertion sites in the N-terminal region (arrowheads). **(D)** Western blots of WT (*cw15*) and *dhc5* (*248, 264, 438, 953*) axonemes probed with different antibodies. N-terminal fragments of DHC5 lacking the motor domain were assembled in all four mutants. No significant defects were noted in assembly of MBO2 or DIC2 (IC69). **(E)** Measurements of forward swimming velocities failed to detect significant differences between WT and the four *dhc5* mutants.

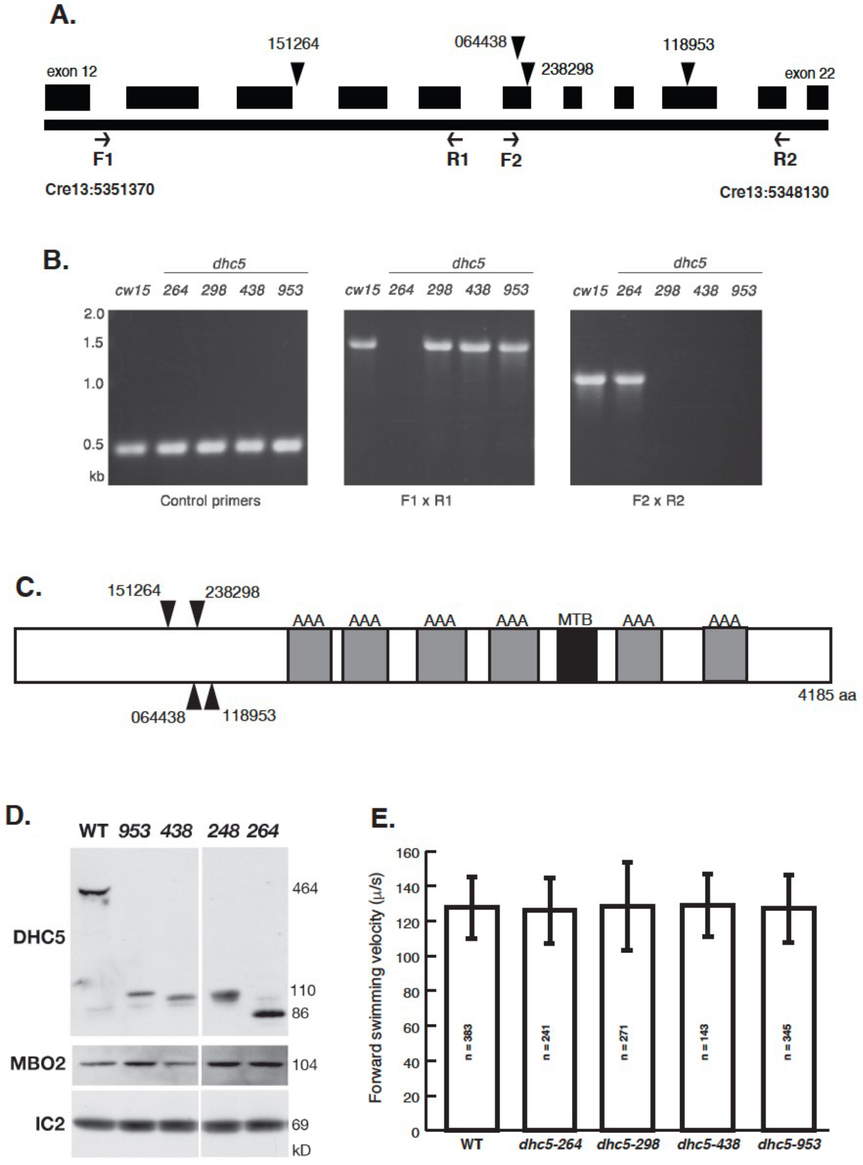

Figure S4. Clustal W alignment of MBO2, FAP58, and FAP189. The full-length polypeptide sequences of MBO2, FAP58, and FAP189 were aligned by Clustal W, with identical amino acids shown in dark gray and similar amino acids in light gray. FAP58 and FAP189 are closely related paralogs.

```
MBO2      1   MDDDILG----LEDVTASEAFKIIYELSKASKIDADRSDAAKAQFTLLHSTLLQALQAERDLLDQAKAL   65
FAP58     1  MASDFSTG-----LHGGHHETLEQ-YNKVLEELAADAVMDPFRVEYEKLHRALRKTYESQARLAKKCQEL   64
FAP189    1  MAEDAGPSKPSEELENRAYEALERDFQEVLQELVGDKSLERFRIEYEKLHRALKKSHESEKRLIKKCREL   70

MBO2     66  KRQKEEQEAAITGAGGVPG--ADDIDQLREDVEAALGEAALAQERQQLLQLEVTDLQRQRNDLGARMEEL  133
FAP58    65  NSDISLNASKVQSALKLNEEDRETAVALKREINKAWKMVDDSTVKETKAKETAQQLKVEIANL-SRLVEE  133
FAP189   71  NAEIVANAAKVQTALKLNEEDQQTIGALKKEIEKAWKMVDASHEKEAKAKETIQQLKIEIANL-TRLVEQ  139

MBO2    134  AAEHAAALQPVIAQARGEAAALVDELDEERRRVEAAHGELEDMKSKLAAVQEEIEAMGETKAVERANLVK  203
FAP58   134  GAGLAIGEETALNELLKQKEELARERDAQVEQLMKYRSDLMETQEKLRAADAEKLQLDADIQHLRGTIND  203
FAP189  140  GAGLAIGEETTLNELLKQKEELVRERDAQVDQIMQLRSDLMEMQEKQRTAEADKLQLDMDIQQLKDSVAE  209

MBO2    204  VDPLPDKARRQSEAVAAILKSVQGQLESMNARYGEHDTQYKTAAEREQQLTEEHTKMLTALERGRVQVES  273
FAP58   204  KKAEAEREIRKKERMEKEMKELRQQLEIRSSEIKSKQLQVTSTEEQVARLEQMLRDAKFATEKVQKEYNM  273
FAP189  210  KKAEAEREIRKKERMEKEMKELRQQLEIRSSEIKTKQLQMTSTEEQVAKLEQMVRDSKAATEKVQKEYNM  279

MBO2    274  KARHADDVRKDVELASIEADKILADQVELDLRVKNLALELKAESDHLNRRQREKELMLRQYRVADQQLKD  343
FAP58   274  LNERMQKLHHDLEEQIHTNTQLLTENSAKQVELRVKEEEISGIKQEASRVNKLREQTVKKTKQLEEQRVE  343
FAP189  280  LNEKMQKLHHDLEEQIHTNTQLLAENSAKQVELKVKDEEIAAIKQEASRVNKLREQTVKKTKQLEEQRTE  349

MBO2    344  ARDMLPNLKFQVEQMHRDVNTLEARRRAQSRELSDVKRELDIQMAAFLHEEADGKEKVALFQLTYKEVAA  413
FAP58   344  VEKERDVLRAELAALERELEAKQKEVDVEKKKLEELTRERDVLTKLRSQAENATQKQIDLVKINENAKRN  413
FAP189  350  VEKERDVLKSELAALERDVEAKQKEVELEKKKLEELMRERDVLTKMRTQAENATQKQTDMIKINENTKRN  419

MBO2    414  LEAELAALKREEAERDTILRDLGSQRDRVALAIAQKLSKVKDVQMTSRIKEVELAELKKIRKEVGRRIRD  483
FAP58   414  LEQEIQGYKMEAQKQSKLIYQLEKEREKYDLEAAEAANKYQQAQSEVKLRVDAIMDLQRRIAEGESKLKQ  483
FAP189  420  LEQEIQGYKTEAQKQSKLIYQLEKEREKYSIEASDASAKYMQALEEVKLREMAIIDLQKRIAEGESKLKQ  489

MBO2    484  FEKLYDLVKNQRNKFVNLIQAASQSTTEMKDKLKVLQNELDILQNEVGIKDKLLQQQHTQHAANIAERDQ  553
FAP58   484  QQNLYEAVRADRNLYSKNLIEAQDEIQEMKRKFKIMQHQIEQLKEEITGKDLYLLKEHFEHQKVINEKEQ  553
FAP189  490  QQNLYEAVRADRNLYSKNLIEAQDEIQEMKRKFKIMQHQIEQLKEEITGKDLYLLKEHFDHQKVIKEKDL  559

MBO2    554  LRVELGRLGMVFRDKQAVVDEQIAEVDKLNAIINGCEKEMLRLKKQYELVIEARNYTGIMLIDRNDELCV  623
FAP58   554  LRNELDRSKSNIKEADSAINAQKVEIDKLNHIINEADQERRRQKKEYDIVVNERDILGTQLVRRNDELAA  623
FAP189  560  LRAELDKSKAQIKEADAAISSQKAEIDKLNHIINEADQERIRQKKEYDIVVNERDILGTQLVRRNDELAL  629

MBO2    624  LYEKANILDEVIKSGQLELMRREDEARLLRLEVGELERSIGVTRRLVPSVPLLDNDVAALQKALFEARRE  693
FAP58   624  LYERIKIQQATLQMGQSQYRDRLAEIRQLKVRLADLKRQLHLLKSSVSNIDVLKREVHQLGRELLQERTK  693
FAP189  630  LYEKIKIQQSTLAKGQIQYRDRLNEIRVLKVKLADLKRELHILKSSVSNIDVLKREVHQLGRELLQERTK  699

MBO2    694  AEALSLALENPSNQGRWRLLEGKIPDREELSAKIQALEERLNDKKEQLLEKELILEEITSLSDKLRVQAA  763
FAP58   694  VKALSEELENPLNVHRWRKLEGSDPGTYEMIQKIQTLQKRLISKTEEVVEKDLLIQEKEKLYMELKNILA  763
FAP189  700  VKALSEELENPLNVHRWRKLEGSDPGTYEMIQKIQTLQKRLISKTEEVVEKDLLIQEKEKLYMELKNILA  769

MBO2    764  EGRADTLELAQRVNEYQSKLRAVTRKIMATVSELSMYQASALKLGAEKEELEGAVSLASQRLEAGEPPTD  833
FAP58   764  R--QPGPEVAEQLSIYQANLREKTKQMKAMASELNMYQAQVNEYKYEIERLVRELNEMKQVFFDRR----  827
FAP189  770  R--QPGPEVAEQLSIYQANLREKTKQMKAMASELNMYQAQVNEYKYEIERLVRELNEMKRKYFEGK----  833

MBO2    834  DAEREWYRLERERHTVDAMAEERRAVAAALDARVAEVQSTAEPRPNAYIPEQLG-IPKPYGSFAPFKPQE  902
FAP58   828  -------------------------------KKEQADRARTMKASMYGPSLLDQLPGGSGTGSGGMATG  865
FAP189  834  -------------------------------RREQMERERTMKPPAPAP------PNASQPR----FTG  861

MBO2    903  AGSTMRHIRKPSPKEVVI  920
FAP58   866  GGVGMS              871
FAP189  862  GGFSLAQ             868
```

Figure S5. The structure of a MBO2-FAP58 heterodimer as predicted by AlphaFold 2.

The potential interactions between the full-length MBO2 and FAP58 polypeptide sequences were analyzed using AlphaFold 2 multimer. The predicted structure of the heterodimer was visualized using PyMol and is shown here. The MBO2 sequence is shown in green, and the FAP58 sequence is shown in red. Both sequences were predicted to contain unstructured regions at their N- and C-termini. The alignment of the coiled coil domains is consistent with recent crosslinking studies indicating extensive interactions between two orthologues of these proteins, CCDC146 and CCDC147, in *Tetrahymena* (McCafferty et al., 2023).

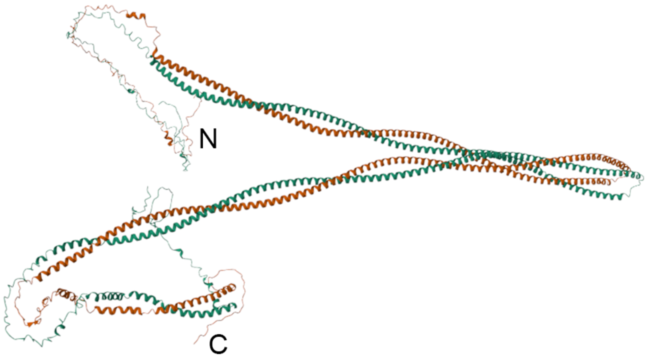

Figure S6. A model illustrating the proposed location of FAP57 relative to other structures in the 96 nm repeat. (A) Tomographic slice through an averaged 96-nm repeat from WT axonemes, showing the region projecting from the surface of the A-tubule above protofilament A04 and containing the ICLC complex of the I1 dynein and the N-DRC. The coiled coil MIA complex extending from the base of the I1 ICLC is indicated in orange. The MIA complex contacts another coiled coil structure (red) on the surface of the DMT corresponding to MBO2/FAP58 (or FAP189). A second filamentous structure predicted to be coiled coil domain of FAP57 is located above the surface of the DMT and indicated in blue. It extends from the base of a FAP57 globular domain to a site beyond the N-DRC, curving down to a cleft between protofilaments A03 and A04 on the right (blue arrowheads). **(B)** Isosurface rendering of the structures shown in (A). The I1 dynein is sliced through one of its motor domains and colored in bright pink. The tether head structure below I1 is colored in magenta, and the bases of the other IDAs are colored in light pink. The CCDC39/40 complex forming the axonemal ruler between protofilaments A02 and A03 is shown in gold. The radial spokes (RS1, RS2, and RS3S) are cut close to their bases and colored in shades of green and light blue. The N-DRC structure is yellow, and structures missing in *mbo2* are colored in red. The blue- labeled structure corresponds to the proposed location of FAP57. Its N-terminal, WD repeat domain is located near the I1 ICLC and MIA complex (Yamamoto et al., 2013; Lin et al., 2019). Its coiled coil domain "floats" above the surface of the DMT, running behind the N-DRC and the "distal staple" (composed of CCDC96 and CCDC113, Bazan et al, 2021) and then curving down towards the bases of IDA *g* and IDA *d.* **(C)** An isosurface rendering of the WT 96 nm repeat with Alphafold2 predicted models of MIA complex (FAP100 and FAP73 in orange) and FAP57 (amino acids 1-771, 810-1050, 1060-1123 in blue) as proposed by Walton et al. (2023) shown relative to our model of the MBO2/FAP58 heterodimer (in red). The C-terminal region of FAP57 (amino acids 1124-1316) is not shown**.** The FAP57 coiled coil domain is proposed to interact with the CCDC96/CCDC113 staple, FAP337, near the base of IDA *g*, and the FAP43/44 subunits of the I1 tether head (Lin et al., 2019; Bazan et al., 2021, Ghanaeian et al., 2023; Walton et al., 2023). Note that *Chlamydomonas* contains three FAP57 paralogues (FAP57, FBB7, and FAP331) and two FAP337 paralogues (FAP337 and Cre07.g313850) (Lin et al., 2019); these three proteins may form homo- or heterodimers that differ between DMTs or proximal/distal regions of the axoneme. Scale bar is 25 nm in (A).

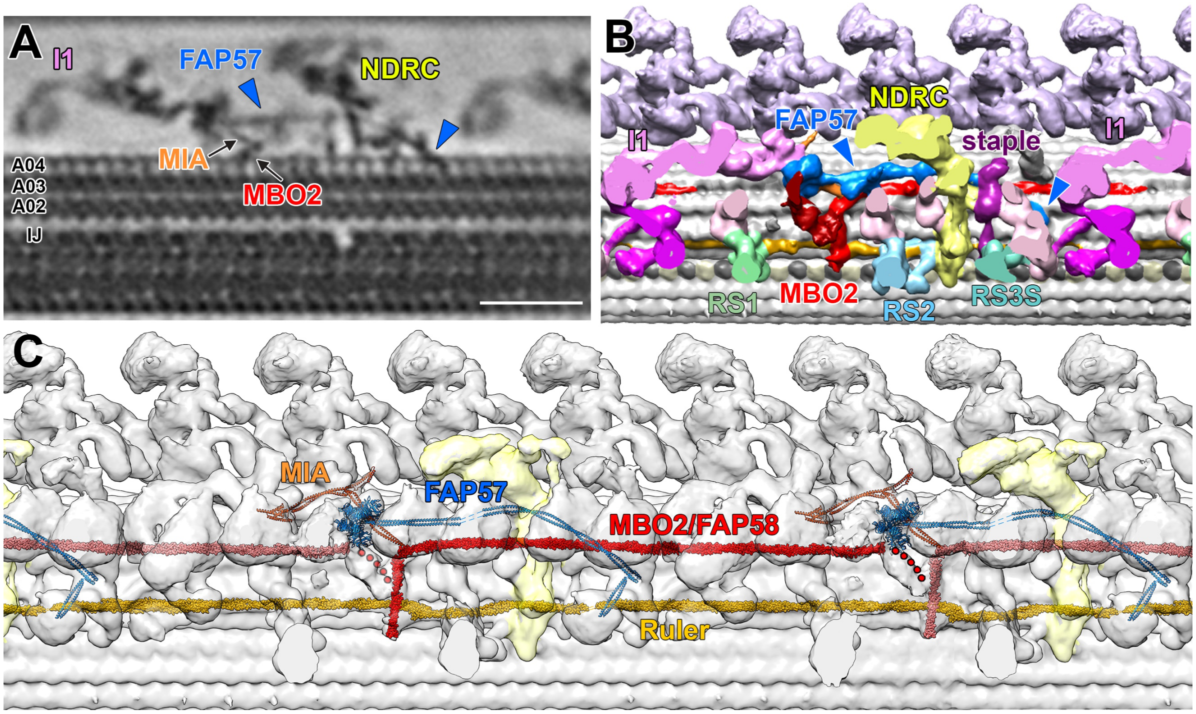

Supplemental Video 1. Visualization of the averaged doublet microtubule from a *Chlamydomonas* WT axoneme. The video was generated using the tomogram obtained from the highest resolution WT axoneme sample (pWT, see Supplemental Table S5). The tomographic slices in cross-sectional orientation (from proximal to distal direction) and longitudinal orientation (moving from the B-tubule through the A-tubule) are displayed at the beginning of the video. The positions and densities corresponding to the ruler and MBO2 structures are indicated by labeling and showing their 3D isosurface renderings. The isosurface rendering of the entire averaged structure is shown in different orientations, with emphasis on the L-shaped MBO2 associated structure. The localizations of the Au-SNAP tags at the N-, M- and C-terminal regions are highlighted by yellow stars, and the flexible N-terminal region of MBO2 that is not visible in the sub-tomogram averages is indicated by a red dotted line.

Supplemental Table 1. Strains used in this study

Strain name	CC number	Motility phenotype	References
**Control strains**			
137c*, mt-* (*nit1; nit2; agg1*)	CC-124	Forward swimming	Harris, 1989
137c*, mt+* (*nit1; nit2*)	CC-125	Forward swimming	Harris, 1989
cw15; mt-	CC-4533	Forward swimming	Li et al., 2016, 2019
**MBO2 related**			
mbo2-4	CC-4190	Move backwards only<br>Symmetric waveform<br>Reduced DMT beaks	Tam and Lefebvre, 2002
mbo2-4; MBO2-HA	CC-5325	Forward swimming	Tam and Lefebvre, 2002; this study
mbo2-4; MBO2-N-SNAP-C-HA	CC-5505	Forward swimming	This study
mbo2-4; MBO2-C-SNAP	CC-5504	Forward swimming	This study
mbo2-4; MBO2-M-SNAP-C-HA	CC-5506	Forward swimming	This study
**Dynein mutants**			
dhc5-264	*(LMJ.RY0402.151264)*	Forward swimming	Li et al., 2016, 2019
dhc5-298	*(LMJ.RY0402.238298)*	Forward swimming	Li et al., 2016, 2019
dhc5-438	*(LMJ.RY0402.064438)*	Forward swimming	Li et al., 2016, 2019
dhc5-953	*(LMJ.RY0402.118953)*	Forward swimming	Li et al., 2016, 2019
ida2	CC-2666	Slow smooth, lacks I1(*f*) IDA	Kamiya et al., 1991
ida4	CC-2670	Slow smooth, lacks IDA *a, c, d*	Kamiya et al., 1991; Kagami & Kamiya, 1992
ida5	CC-3420	Slow smooth, lacks IDA *a, c, d, e*	Kato et al., 1993; 1997
IO	CC-2012	Paralyzed, lacks ODA and IDA	
pf28	CC-1877	Reduced frequency, lacks ODA	Mitchell & Rosenbaum, 1985
**Other motility mutants**			
ida8-1 (fap57)	CC-4089	Slow swimmer, suppressor of *pf10*<br>FAP57, FAP337 reduced<br>FBB7, FAP331, Cre07.g313850 increased	Dutcher et al., 1988; Lin et al., 2019
mbo1	CC-2679	Moves backwards only<br>Symmetric waveform<br>Reduced DMT beaks	Segal et al., 1984
mbo3	CC-2378	Mixed *mbo* and twitchy	Segal et al., 1984; Tam and Lefebvre, 2002
pf10	CC-1296	Symmetric waveform<br>Forward, jiggling movement	Ramanis & Luck, 1986; Dutcher et al., 1988
pf12 (pacrg)	CC-1031	Symmetric waveform<br>Slow forward movement<br>Reduced DMT beaks, IDA *b*<br>Defective inner DMT junction	McVittie, 1972; Tam & Lefebvre, 2002; Dymek et al., 2019
pf12; fap20	CC-5582	Symmetric waveform<br>Slow forward movement<br>Reduced DMT beaks, IDA *b*<br>Missing inner DMT junction	Dymek et al., 2019
pf14	CC-1032	Paralyzed, lacks radial spokes	Luck et al., 1977
pf9-2; pf28	CC-3900	Paralyzed, short flagella<br>Lacks ODA, I1 dynein<br>DHC3, DHC4, DHC11 increased	Porter et al., 1992; Hwang et al., 2024

Supplemental Table 2. Oligonucleotide sequences used in the study

Name and purpose	Nucleotide sequence
**PCR of *dhc5* insertions**	
Control primers (498 bp)	5'-CAGTCATCACAACACTCATCG-3'<br>5'-GCTGCGGAAGACTCACTTAG-3'
DHC5, F1 x R1 (1440 bp)	5'-GGGGTAACCACTACAAGCCC-3'<br>5'-GACTTACCTTGAGCGCCTT-3'
DHC5, F2 x R2 (1073 bp)	5'-ACGAGTTCCAGAAGGTGCTG-3'<br>5'-CCAGCCGTACCTGAACTTGT-3'
**Mutagenesis and epitope tagging of *MBO2***	
PCR of *N-SNAP-MBO2* fragment (*Apa*I)	5'-GCCCTTGTTAGCgggcccAATACCACCGGCCGCGTGC-3'<br>5'-CGGTATTAATGACCAGCAGgggcccGACCGCAAGCATG-3'
PCR of central *MBO2* fragment	5'-AGAACGAGGTGGGCATCAAG-3'<br>5'-CAGGGATGTAGGCATTGGGG-3'
Site-directed mutagenesis of *MBO2* fragment (*Hind*III)	5'-CGCGACaagcttGCGGTGGTGG<br>5'-GAACACCATGCCAAGCCTG
PCR of SNAP for insertion into *Hind*III	5'-<br>TGGCATGGTGTTCCGCGACaagcttATGGACAAGGACTGCGAGATGAAGC<br>GCAC-3'<br>5'-tctgctcgtccaccaccgcaccGGAGCCCGGGGCGCCGAT-3'
PCR of *MBO2-M-SNAP* fragment (*Kpn*I)	5'-TGGGGTAGCTATCAggtaccGCTGCGGTAGTGGTGCTC-3'<br>5'-CCGGGGGACGACGAggtaccAGTATTTTACATCGTGCTCCATAAC-3'
PCR of *MBO2-C-SNAP* fragment (*Hpa*I)	5'- AGCTGCTCTTGGgttaacAGCTGGCAGTTTAGC-3'<br>5'- AGTTCACGGCTgttaacGTAACACAGGTTCACC-3'

The regions containing the predicted sites of plasmid insertion in the *dhc5* mutants were amplified using the FailSafe PCR enzyme and buffer G (Illumina, San Diego, CA). The regions encoding the *N-SNAP-MBO2* and *MBO2-C-SNAP* gene fragments were synthesized by Genewiz (Azenta, Carol Stream, IL) and amplified using the CloneAmp enzyme (Takara, San Jose, CA) for subcloning into the full-length *MBO2* gene at the appropriate restriction sites indicated in lower case. For tagging the middle region of the *MBO2* gene, a fragment spanning two *Kpn*I sites was amplified using the FailSafe PCR enzyme and buffer G, subcloned into pGEM, and subjected to site-directed mutagenesis to create a *Hind*III site. The SNAP tag was amplified using the CloneAmp enzyme and cloned into the *Hind*III site of the *MBO2* fragment. The *MBO2-M-SNAP* fragment was then re-amplified with the CloneAmp enzyme for subcloning into the full-length *MBO2* gene. The predicted amino acid sequences of the tagged MBO2 polypeptides are shown in Supplemental Figure S1.

Supplemental Table 3. Antibodies used in this study

Antigen	Host	Dilution (WB)	Reference or source
DRC1	Rabbit	1:1000-1:10000	Wirschell et al., 2013
Gas8 fusion (DRC4)	Rabbit	1:1000-1:10000	Bower et al., 2013
CCDC39/FAP59	Rabbit	1:1000	Sigma #HPA035364
FAP57	Rabbit	1:1000-5000	This study
Rib43	Rabbit	1:10000-1:20000	Norrander et al., 2000
Rib72	Rabbit	1:10000-1:20000	Ikeda et al., 2003
tektin	Rabbit	1:20000	Yanagisawa & Kamiya, 2004
DHC5	Rabbit	1:1000	Yagi et al., 2009
DHC9	Rabbit	1:5000	Yagi et al., 2009
IC140	Rabbit	1:10000	Yang & Sale, 1998
IC2 (IC69)	Mouse	1:10000-1:20000	Sigma #D6168
IC138	Rabbit	1:10000	Hendrickson et al., 2004
MIA1	Rabbit	1:1000	Yamamoto et al., 2012
MIA2	Rabbit	1:1000	Yamamoto et al., 2012
MBO2	Rabbit	1:1000	Tam and Lefebvre, 2002
RSP16	Rabbit	1:10000-1:20000	Yang et al., 2005
HA (3F10)	Rat	1:500-1000	Roche #1867423
GFP	Mouse	1:5000	Covance #MMS-118P
SNAP	Rabbit	1:1000	New England Biolabs #P9310S

Supplemental Table S5. Summary of image processing information for strains used in this study

Strains	Tomogram numbers (proximal/distal)	Averaged DMT repeats	TEM/ camera	Resolution (nm)[a]	Usage in Figures
WT	25 (5/20)	3736	F30/CCD	3.8	Figure 3A-D, I-K<br>Figure 4, Figure 6E, F<br>Figure 7E,<br>Figure 5A, C, E
WT	19 (6/13)	2381	Krios/K2	2.3	Figure 7A, C,<br>Figure 8A, B,
pWT[b]	85 (26/59)	11519	Krios/K2	1.8	Figure 9, Figure S6<br>Figure 5G-J
mbo2	37 (10/27)	4525	F30/CCD	3.1	Figure 3E-H, I-K<br>Figure 4, Figure 6E, F<br>Figure 5B, D, F
mbo2; MBO2::SNAP (C)	14 (4/10)	1898	F30/CCD	3.7	Figure 6E, F, Figure 7F
mbo2; SNAP::MBO2 (N)	14 (5/9)	2257	Krios/K2	2.4	Figure 6E, F, Figure 7B
mbo2; MBO2::SNAP (M)	25 (7/18)	3154	Krios/K2	2.5	Figure 6E, F, Figure 7D

[a] Resolution based on the 0.5 criterion of the Fourier shell correlation

[b] Strains include WT, *fap76-1*, *fap81*, *fap92* and *fap76; fap81*, which were used to analyze central apparatus. The central apparatus mutants had WT-like 96-nm axonemal DMT repeats (Fu et al., 2019).

Supplemental references

Bazan, R., Schröfel, A., Joachimiak, E., Poprzeczko, M., Pigino, G., and Wloga, D. 2021. Ccdc113/Ccdc96 complex, a novel regulator of ciliary beating that connects radial spoke 3 to dynein g and the nexin link. *PLoS Genet* 17, e1009388.

Bower, R., Tritschler, D., Vanderwaal, K., Perrone, C.A., Mueller, J., Fox, L., Sale, W.S., and Porter, M.E. 2013. The N-DRC forms a conserved biochemical complex that maintains outer doublet alignment and limits microtubule sliding in motile axonemes. *Mol. Biol. Cell.* 24, 1134-1152.

Dutcher, S.K., Gibbons, W., and Inwood, W.B. 1988. A genetic analysis of suppressors of the *PF10* mutation in *Chlamydomonas reinhardtii. Genetics.* 120, 965-976.

Dymek, E.E., Lin, J., Fu, G., Porter, M.E., Nicastro, D., and Smith, E.F. 2019. PACRG and FAP20 form the inner junction of axonemal doublet microtubules and regulate ciliary motility. *Mol. Biol. Cell.* 30, 1805-1816.

Fu, G., Zhao, L., Dymek, E., Hou, Y., Song, K., Phan, N., Shang, Z., Smith, E.F., Witman, G.B., and Nicastro, D. 2019. Structural organization of the C1a-e-c supercomplex within the ciliary central apparatus. *J. Cell Biol.* 218, 4236-4251.

Ghanaeian, A., Majhi, S., McCaffrey, C.L., Nami, B., Black, C.S., Yang, S.K., Legal, T., Papoulas, O., Janowska, M., Valente-Paterno, M., Marcotte, E.M., Wloga, D., and Bui, K.H. (2023). Integrated modeling of the Nexin-dynein regulatory complex reveals its regulatory mechanism. *Nat Commun*. 14:5741. doi: 10.1038/s41467-023-41480-7.

Harris, E. 1989. The *Chlamydomonas* Sourcebook. Academic Press: San Diego, CA. 780 pp

Hendrickson, T.W., Perrone, C.A., Griffin, P., Wuichet, K., Mueller, J., Yang, P., Porter, M.E., and Sale, W.S. 2004. IC138 is a WD-repeat dynein intermediate chain required for light chain assembly and regulation of flagellar bending. *Mol. Biol. Cell.* 15, 5431-5442.

Hwang, J., Yanagisawa, H., Davis, K.C., Hunter, E.L., Fox, L.A., Jiminez, A.R., Goodwin, R.E., Gordon, S.A., Stuart, C.D.E., Bower, R., Porter, M.E., Dutcher, S.K., Sale, W.S., Lechtreck, K.F., and Alford, L.M. (2024). Assembly of FAP93 at the proximal axoneme in *Chlamydomonas*. *Cytoskeleton* doi: 10.1002/cm.21818.

Ikeda, K., Brown, J.A., Yagi, T., Norrander, J.M., Hirono, M., Eccleston, E., Kamiya, R., and Linck, R.W. 2003. Rib72, a conserved protein associated with the ribbon compartment of flagellar A-microtubules and potentially involved in the linkage between outer doublet microtubules. *J. Biol. Chem.* 278, 7725-7734.

Kagami, O., and Kamiya, R. 1992. Translocation and rotation of microtubules caused by multiple species of *Chlamydomonas* inner-arm dynein. *J. Cell Sci.* 103, 653-664.

Kamiya, R., Kurimoto, E., and Muto, E. 1991. Two types of *Chlamydomonas* flagellar mutants missing different components of inner-arm dynein. *J. Cell Biol.*112, 441-447.

Kato, T., Kagami, O., Yagi, T., and Kamiya, R. 1993. Isolation of two species of *Chlamydomonas reinhardtii* flagellar mutants, *ida5* and *ida6*, that lack a newly identified heavy chain of the inner dynein arm. *Cell Struct. Funct.* 18, 371-377.

Kato-Minoura, T., Hirono, M., and Kamiya, R. 1997. *Chlamydomonas* inner-arm dynein mutant, *ida5*, has a mutation in an actin-encoding gene. *J. Cell Biol.*137, 649-656.

Li, X., Patena, W., Fauser, F., Jinkerson, R.E., Saroussi, S., Meyer, M.T., Ivanova, N., Robertson, J.M., Yue, R., Zhang, R., Vilarrasa-Blasi, J., Wittkopp, T.M., Ramundo, S., Blum, S.R., Goh, A., Laudon, M., Srikumar, T., Lefebvre, P.A., Grossman, A.R., and Jonikas, M.C. 2019. A genome-wide algal mutant library and functional screen identifies genes required for eukaryotic photosynthesis. *Nat Genet.* 51, 627-635.

Li, X., Zhang, R., Patena, W., Gang, S.S., Blum, S.R., Ivanova, N., Yue, R., Robertson, J.M., Lefebvre, P.A., Fitz-Gibbon, S.T., Grossman, A.R., and Jonikas, M.C. 2016. An Indexed, Mapped Mutant Library Enables Reverse Genetics Studies of Biological Processes in *Chlamydomonas reinhardtii. Plant Cell* 28, 367-387.

Lin, J., Le, T.V., Augspurger, K., Tritschler, D., Bower, R., Fu, G., Perrone, C., O'Toole, E., Mills, K.V., Dymek, E., Smith, E.F., Nicastro, D., and Porter, M.E. (2019). FAP57/WDR65 targets assembly of a subset of inner arm dyneins and connects to regulatory hubs in cilia.

Luck, D., Piperno, G., Ramanis, Z., and Huang, B. 1977. Flagellar mutants of *Chlamydomonas*: studies of radial spoke-defective strains by dikaryon and revertant analysis. *Proc. Natl. Acad. Sci. U.S.A.* 74, 3456-3460.

McVittie, A. 1972. Flagellar mutants of *Chlamydomonas reinhardtii. J. Gen. Microbiol.* 71, 525-540.

Mirdita, M., Schütze, K., Moriwaki, Y., Heo, L., Ovchinnikov, S., and Steinegger, M. 2022. ColabFold: making protein folding accessible to all. *Nat. Methods.* 19, 679-682.

Mitchell, D.R., and Rosenbaum, J.L. 1985. A motile *Chlamydomonas* flagellar mutant that lacks outer dynein arms. *J. Cell Biol.* 100, 1228-1234.

Norrander, J.M., deCathelineau, A.M., Brown, J.A., Porter, M.E., and Linck, R.W. 2000. The Rib43a protein is associated with forming the specialized protofilament ribbons of flagellar microtubules in *Chlamydomonas*. *Mol. Biol. Cell.* 11, 201-215.

Pazour, G.J., Agrin, N., Leszyk, J., and Witman, G.B. (2005). Proteomic analysis of a eukaryotic cilium. J Cell Biol *170*, 103-113.

Porter, M.E., Power, J., and Dutcher, S.K. (1992). Extragenic suppressors of paralyzed flagellar mutations in *Chlamydomonas reinhardtii* identify loci that alter the inner dynein arms. J Cell Biol *118*, 1163-1176.

Ramanis, Z., and Luck, D.J. 1986. Loci affecting flagellar assembly and function map to an unusual linkage group in *Chlamydomonas reinhardtii. Proc. Natl. Acad. Sci. U.S.A.* 83, 423-426.

Sakato-Antoku, M., and King, S.M. (2022). Developmental changes in ciliary composition during gametogenesis in. Mol Biol Cell *33*, br10.

Segal, R.A., Huang, B., Ramanis, Z., and Luck, D.J.L. 1984. Mutant strains of *Chlamydomonas reinhardtii* that move backwards only. *J. Cell Biol.* 98, 2026-2034.

Tam, L.W., and Lefebvre, P.A. 2002. The *Chlamydomonas MBO2* locus encodes a conserved coiled-coil protein important for flagellar waveform conversion. *Cell Motil. Cytoskeleton.* 51, 197-212.

Walton, T., Gui, M., Velkova, S., Fassad, M.R., Hirst, R.A., Haarman, E., O'Callaghan, C., Bottier, M., Burgoyne, T., Mitchison, H.M., and Brown, A. (2023). Axonemal structures reveal mechanoregulatory and disease mechanisms. Nature *618*, 625-633.

Wirschell, M., Olbrich, H., Werner, C., Tritschler, D., Bower, R., Sale, W.S., Loges, N.T., Pennekamp, P., Lindberg, S., Stenram, U., Carlen, B., Horak, E., Kohler, G., Nurnberg, P., Nurnberg, G., Porter, M.E., and Omran, H. 2013. The nexin-dynein regulatory complex subunit DRC1 is essential for motile cilia function in algae and humans*. Nat Genet.* 45, 262-268.

Yagi, T., Uematsu, K., Liu, Z., and Kamiya, R. (2009). Identification of dyneins that localize exclusively to the proximal portion of *Chlamydomonas* flagella. J Cell Sci *122*, 1306-1314.

Yamamoto, R., Song, K., Yanagisawa, H.A., Fox, L., Yagi, T., Wirschell, M., Hirono, M., Kamiya, R., Nicastro, D., and Sale, W.S. 2013. The MIA complex is a conserved and novel dynein regulator essential for normal ciliary motility. *J. Cell Biol.* 201, 263-278.

Yanagisawa, H.A., and Kamiya, R. 2004. A tektin homologue is decreased in *Chlamydomonas* mutants lacking an axonemal inner-arm dynein. *Mol. Biol. Cell.* 15, 2105-2115.

Yang, C., Compton, M.M., and Yang, P. 2005. Dimeric novel HSP40 is incorporated into the radial spoke complex during the assembly process in flagella. *Mol. Biol. Cell.* 16, 637-648.

Yang, P., and Sale, W.S. 1998. The Mr 140,000 intermediate chain of *Chlamydomonas* flagellar inner arm dynein is a WD-repeat protein implicated in dynein arm anchoring. *Mol Biol Cell* 9, 3335-3349.
